# Bacteriophages in gut metagenomes: from analysis to application

**DOI:** 10.1186/s12985-026-03069-6

**Published:** 2026-01-22

**Authors:** Natalia Zakharevich, Aleksandra Strokach, Egor Shitikov, Ksenia Klimina

**Affiliations:** 1 Department of Biomedicine and Genomics, Lopukhin Federal Research and Clinical Center of Physical-Chemical Medicine of Federal Medical Biological Agency, Moscow, Russia; 2 Department of Immunology, Faculty of Biology, Lomonosov Moscow State University, Moscow, Russia

**Keywords:** Bacteriophages, Metagenomes, Gut virome, Gut microbiota

## Abstract

Bacteriophages constitute a major component of the human gut virome, playing very important roles in shaping of the structure and function of the gut microbiota. Moreover, bacteriophages interact with the human immune system, thereby influencing various disease processes. Recent advancements in metagenomic sequencing and computational analysis have substantially expanded our understanding of gut phage diversity and the scale of the so-called ‘viral dark matter’. In this review, we summarize current bioinformatic approaches for identifying and annotating bacteriophage sequences in metagenomic data, discuss key challenges in taxonomic classification and host prediction of phages, as well as the limitations associated with the assembly and analysis of viral metagenome-assembled genomes (vMAGs). We also analyze the therapeutic potential of bacteriophages, including their application in cancer immunotherapy, inflammatory diseases, and liver diseases, and their promise as diagnostic and prognostic biomarkers.

**Supplementary Information:**

The online version contains supplementary material available at 10.1186/s12985-026-03069-6.

## Introduction

Viruses inhabiting the human body form a special and still insufficiently studied component of the microbiota – the virome. The human virome comprises of eukaryotic viruses, including pathogenic viruses that infect human cells, and viruses that infect bacteria, known as bacteriophages or phages. Plant viruses may also be present in the virome, likely originating from food sources [1, 2]. Viromes vary depending on the biological niche (such as skin, nasopharynx, gut, or urogenital tract), as well as in their impact on human health. In recent years, interest in the virome has grown significantly, driven by advances in metagenomic sequencing and bioinformatic analysis. However, a large proportion of viral sequences detected in human samples – from ~ 70% to ~ 95% – belong to the hidden expanse of viral diversity termed ‘viral dark matter’ [3–10]. Essentially, viral dark matter is the collection of viral sequences retrieved from sequencing data that remain entirely uncharacterized for now: they cannot be taxonomically assigned to established families, genera, or species; their putative hosts are undetermined; and their biological functions remain obscure [11–15].

The gut virome (GV) plays an important role in shaping the composition and function of the human gut microbiota (GM) in both health and in disease. Bacteriophages are predominant in the GV, accounting for up to 90% of its viral component. Phages influence GM diversity and contribute to horizontal gene transfer, thereby shaping and expanding the functional potential of the microbial community [3, 16, 17]. In addition to their direct impact on gut bacteria, there is growing evidence that phages can influence the human body, including the immune system. One such mechanism involves the release of pro-inflammatory molecules into the gut lumen when bacterial cells are lysed by phages. Additionally, evidence of phage transcytosis across gut epithelial cells suggests that phages may directly stimulate the human immune system [18–20]. It is also important to note that phages can significantly reduce the bacterial load and ameliorate disease by specifically targeting pathogenic bacteria in the infectious setting and/or dysbiosis, as has already been demonstrated in a number of animal models [21, 22]. Of particular interest is the ability of phages to enhance the immune response. For instance, it has been shown that a prophage of *Enterococcus hirae* can induce T-cells that cross-react with tumor antigens, thereby increasing tumor sensitivity to immunotherapy [23, 24]. These findings open the possibility of using specific phages to stimulate the immune system in order to improve the effectiveness of immunotherapy and increase the number of patients who respond to it.

Besides to modulating the host immune system, phages can also influence brain function and memory [25, 26]. For example, it was discovered that bacteriophages from the *Demerecviridae* and *Drexlerviridae* families, class *Caudoviricetes* (the original publication uses the now-obsolete order name *Caudovirales* and family name *Siphoviridae*), have been associated with increased memory capabilities through upregulation of the expression of genes involved in neuroplasticity, neuronal development and memory formation [26]. Furthermore, Wu et al. reported a potential role for gut phages in the development of depression, suggesting that phage imbalance may serve as both a distinguishing feature of and a diagnostic marker for the disorder [25].

One of the most striking discoveries of recent years is that the human GV exhibits not only exceptional diversity but also a high degree of individual specificity and temporal stability [2]. Although bacteriophages constitute the majority of viral particles in the gut, their composition is unique to each individual and remains remarkably stable over the course of months. Thus, the phage composition may serve as an individual ‘viral fingerprint’, potentially useful for diagnosing and monitoring changes in the GM. Although much of the genomic diversity of gut phages is still hidden from us in the ‘viral dark matter’, but its contours are gradually becoming clear: exemplified by the two most common fecal phage clades, crAssphage and Gubaphage, identified only in 2014 and 2021 [9, 13], respectively, which together highlight the extent of the gut’s still unexplored viral diversity.

In this review, we consider current bioinformatic approaches to GV analysis using metagenomic data, and discuss the therapeutic potential of bacteriophages, particularly in the context of cancer treatment. Considering that the composition of the phage community appears to be linked to clinical outcomes and reflects the functional state of the GM and the patient’s immune system, we also discuss the potential of phages as biomarkers for predicting response to immunotherapy.

### Identification of bacteriophages in metagenomic data

#### From culturing to bioinformatics

Historically, the identification and characterization of bacteriophages – like bacteria – relied on isolation and culturing techniques [27–31]. However, a significant proportion of bacteria and their viruses remain uncultured under laboratory conditions [32]. The development of next-generation sequencing has facilitated the acquisition of metagenomic data, enabling the direct analysis of all genetic material in a sample, regardless of the possibility of culturing.

In metagenomic research, one can either sequence the entire community and subsequently separate viral sequences using bioinformatic methods, or perform physical separation of the viral and bacterial fractions prior to library preparation to obtain a metavirome. The latter approach risks losing a significant proportion of phages due to their association with the cellular fraction. This occurs because phages may integrate into bacterial genomes as prophages, attach to the cell surface, or be involved in ongoing lysogenic infections. Purification methods can also remove certain groups of phages: for instance, chloroform treatment has been shown to inactivate lipid-containing bacteriophages, filamentous phages, and a number of other tail phages [33, 34]. Whole-community metagenomic sequencing not only enables the identification of viral sequences, but also provides the opportunity to study phage-host interactions. This is particularly relevant for analyzing the GV because current data indicate that temperate phages predominate in its composition [35, 36].

Recent decades have seen significant progress in both isolation, and culturing protocols and bioinformatic methods for virome analysis. To describe and understand bacteriophage population dynamics, their interactions with GM, and ultimately, their impact on human health, global phage diversity is currently being studied mainly in silico, using culture-independent tools that analyze the whole gut community.

#### Computational strategies for identifying viral sequences in metagenomic data

Detection bacteriophages in metagenomic data is a significant challenge. Identification and taxonomic classification of phage sequences are complicated by the absence of a universal marker gene – analog of the 16 S rRNA gene in prokaryotes. Furthermore, potential misclassification of viral sequences as bacterial due to their ability to integrate into the host genome. Mosaicism is a distinctive feature of bacteriophage genomes and results from the dominant role that horizontal gene transfer plays in shaping the architecture of these genomes. Mosaicism is certainly not limited to phage genomes, as bacteria also acquire DNA through horizontal gene transfer, but its scale and impact on bacteriophage evolution are much greater. This leads to two difficulties. First, this leads to assembly problems: traditional methods designed for more stable bacterial genomes often cannot correctly assemble the mosaic sequences of phages, resulting in fragmented or chimeric assemblies. Second, high mosaicism also hinders direct genomic alignment and the construction of correct phylogenetic trees. At a minimum, a balance between sensitivity and speed is necessary to address these challenges. High sensitivity is essential for successfully overcoming the high diversity and rapid evolution of phages, as well as the typically low and uneven coverage of viral sequences in metagenome. Speed is required for processing large data [37–42].

In recent years, advancements in sequencing technologies and metagenomic methods, coupled with the emergence and application of machine and deep learning approaches, have led to the development of a wide range of tools for identifying phage sequences. These tools can be categorized into two main groups: (i) reference-dependent and (ii) reference-independent approaches [8, 43].

(i) Reference-dependent approaches identify viral sequences by aligning them to reference databases. Also, we include reference-dependent k-mer classifiers that search for exact/almost exact matches of k-mers with the database (when installing them, the user must download the database) in this group. These methods demonstrate high accuracy when applied to whole-genome sequencing data and perform well with data obtained by long-read sequencing platforms such as Oxford Nanopore and Pacific Biosciences. However, their performance decreases when processing contigs derived from metagenomic data because they are not adapted to recognize incomplete or short/fragmented sequences [42, 44, 45]. Moreover, their accuracy is limited by the completeness and quality of reference databases, making it challenging to detect novel phages that diverge significantly in sequence from phages from known viral families [46–51]. Such approaches can also be called database-dependent.
(ii) Reference-independent approaches represent an alternative that does not rely on reference databases. These methods can be divided into two subgroups. The first includes tools that analyze gene composition and structural features of the genome, such as GC content, codon usage, gene density, etc., to distinguish between viral and bacterial sequences. These features are either analyzed statistically or utilized as input for machine learning classifiers, as in VIBRANT tool [50]. The second subgroup focuses on analyzing frequency-based characteristics of nucleotide sequences (k-mers) that are specific to viral and bacterial genomes. These features are used for training machine and deep learning classifiers. Machine learning models employ a variety of algorithms, ranging from traditional approaches such as support vector machines, logistic regression, random forests, and naive Bayes to deep learning architectures, including neural networks [42, 44, 45, 48, 50–53].

It should be noted that deep learning techniques are successfully employed nowadays to solve a variety of problems in computational biology, and that deep learning is one of the advanced machine learning algorithms. Examples of tools utilizing deep learning methods are DeepVirFinder (https://github.com/jessieren/DeepVirFinder) and Seeker (https://github.com/gussow/seeker) [45, 53]. DeepVirFinder trained on a large number of sequences and applies convolutional neural networks (CNNs) to search viral genomic signatures, which are then used to construct a predictive model capable of determining whether a given sequence is of viral origin. Employing long short-term memory (LSTM) models and a type of recurrent neural network (RNN), Seeker enables rapid phage detection in metagenomic datasets by differentiating phage sequences from bacterial sequences, even when phage sequences exhibit minimal similarity with known phage families. This tool can be said to be less biased: it doesn’t rely on predefined sequence features. Instead, it is trained to read the entire DNA sequence and evaluate the likelihood of their belonging to phage genomes.

Experience shows that reference-dependent tools exhibit a low level of false positives. At the same time, reference-independent (in particular machine and deep learning tools) approaches demonstrate greater sensitivity to phages with lower representation [8, 45]. Furthermore, reference-independent tools can work with shorter contigs compared to reference-dependent tools, as they do not require, for example, the presence of several genes to classify sequences. Most importantly, reference-independent tools are capable of identifying novel phage sequences – part of the so-called ‘viral dark matter’ – unlike reference database dependent methods. However, it is worth noting that machine learning tools still depend to some extent on reference databases, as these are used to train the classifiers. In addition, long-read sequencing (which was already mentioned above (i)) represents a promising complement to short-read sequencing. For instance, it can be used to reconstruct megaphage genomes and obtain information about methylation patterns – which can aid in host prediction, as well as to study population structure at the level of an individual virions. In some cases, long-reads may span complete or nearly complete viral genomes [54].

In recent years, hybrid approaches that combine elements of reference-dependent and -independent methods have been actively developed. For example, VIBRANT (https://github.com/AnantharamanLab/VIBRANT) – hybridizes neural network machine learning and protein signatures, and geNomad (https://github.com/apcamargo/genomad) – combines machine learning with protein profile databases to improve prediction accuracy [50, 55].

The choice of tool for analyzing metagenomic data significantly influences the predicted viral community composition. Different computational approaches often yield divergent results; for instance, comparisons of predicted viral sequences show that the overlap between any two tools rarely exceeds 40% [8]. This highlights the need for a multi-tool strategy for predicting viral genomes in a metagenomic context, where the combination of different algorithms can provide a more reliable consensus. In Table 1, we compiled information on phage detection tools in metagenomic data published over the past decade, and also tried to briefly describe their strengths and limitations [44–53]. In addition, we would like to provide a brief overview of existing virus/bacteriophage databases – Supplementary Table 1 provides their description, content and some notes.

**Table 1:** An overview of available published metagenomic phage detection tools over the past decade

Tool/ year/ reference	Based on/ group	Strengths and limitations	Repository
VirSorter 2015 [46]	viral sequences prediction using probabilistic models and using reference-guided, gene-content based rules **(i)**	• One of the first automated pipelines for finding viral sequences in metagenomic data • Able to classify prophage sequences; also predicts lytic phages; assigns a confidence category (1–3) (the algorithm is good at finding prophages, but was less successful at detecting new free-living viruses) • Runs slowly on large metagenomic datasets compared to modern tools • VirSorter do not classify fragments shorter than 1000 nucleotides (nt); • Effect of contig length on performance: strongly affected by fragment length, with performance a marginal increase with increasing length; performed well for longer contigs • Decrease in precision due to low viral abundance; • Demonstrate low false positive rates and robustness to eukaryotic contamination (as all tools that use a homology approach)	https://github.com/simroux/VirSorter
MetaPhainder 2016 [47]	viral sequences prediction using integrated analysis of BLASTn hits to a phage database **(i)**	• Account for the mosaic genome: taking into account all hits (not the best hit) to the phage database is advantageous when dealing with mosaic phage genomes • Effect of contig length: fragment length largely unaffected on tool performance; demonstrate fairly consistent sensitivity and precision (interestingly, a homology-based tool exhibits a pattern similar to the sequence-based tools for this property) • Has a higher sensitivity, including to phages with less representation in reference databases; decrease in precision due to low viral abundance • Eukaryotic contamination: show much lower specificity, and frequently misclassifying eukaryotic fragments as viral, with an FPR around 0.5 • CheckV evaluate: contain a low proportion of contigs with Medium Quality or higher, suggesting a potential number of FPs • Using BLASTn and calculating ANI requires significant computational resources, especially on large datasets - a slower approach	https://github.com/vanessajurtz/MetaPhinder
Marvel 2017 [48]	viral sequences prediction using a random forest machine learning approach and three simple genomic features **(i)+(ii)**	• The first tool capable to effectively separate metagenomic bins containing dsDNA phage sequences from those containing bacterial sequences (as opposed to treating contigs as isolated objects) • Uses a machine learning approach and three simple genomic features extracted from contig sequences: gene density, strand shifts, and fraction of significant hits against pVOGs database • Requires bins as input • Demonstrate good performance on the recall (sensitivity) measure • The tool’s performance is less tightly coupled to contig length: shows more variability in sensitivity for a given length, since the tool performs classification of contigs after binning, and contigs of various lengths may be present in the same bin • CheckV evaluate: does not predict contigs below medium quality	https://github.com/LaboratorioBioinformatica/ MARVEL
viralVerify 2020 [49]	viral sequences prediction using a Naive Bayesian classifier **(i)**	• Can be used as a standalone tool to predict contigs of viral origin in any assembled metagenome, or as a separate module (MetaviralSPAdes, viralFlye) • Supports creation of a custom training database (from viral, chromosomal and plasmid contigs) and working with a custom HMM database • High precision but have length-dependent sensitivity: performance increasing with length • Robustness to low viral abundance in the community, especially on shorter contigs (about 500 nt) • Gene content dependence • Lack of independent comparisons: viralVerify has not participated in any current major benchmarks	https://github.com/ablab/viralVerify
VIBRANT 2020 [50]	viral sequences prediction using hybrid neural networks of protein annotation signatures **(i)+(ii)**	• Recovery of both free and integrated viral genomes from metagenomes • Hybridizes neural network machine learning and protein signatures • Software that integrates: virus identification, annotation, estimation of genome completeness and distinguishing between lytic and lysogenic viruses, and is also capable of assembling useful annotation data and categorizing the metabolic pathways of viral AMGs • Utilizes metrics of non-reference-based protein similarity annotation databases in conjunction with a unique “v-score” metric - give a quantifiable value to viral hallmark genes instead of categorizing them in a binary fashion • Prediction on short sequences: does not consider scaffolds shorter than 1000 bp or those that encode less than four predicted open reading frames • Reduced sensitivity on short contigs (< 3 kb): exhibits improved performance with scaffolds at least 3 kb in length • Eukaryotic DNA: VIBRANT effectively filtered out eukaryotic contamination by assigning them low v-scores, successfully recovering few false-positive viral sequences • May miss highly novel or divergent viruses	https://github.com/AnantharamanLab/VIBRANT
VirSorter2 2021 [51]	viral sequences prediction using random forest classifiers using an hmmsearch of predicted genes **(i)+(ii)**	• Memory usage stays nearly constant with increasing data size, and scales nearly linearly with threads used • Written with snakemake: total CPU time is higher than other tools, but internal processes are highly parallelizable; this increased CPU time is mostly due to the annotation step (> 90% of CPU time) • Can be scaled to handle large-scale (> 100,000 sequences) datasets • Through modular framework: has a uniquely able to reliably detect different types of non-Caudoviricetes viruses; also, modular framework to enable modification and addition of new classifiers as our knowledge of viral sequence space increases • Users can omit the NCLDV, *Lavidaviridae*, and RNA virus classifiers for analysing bacterial and archaeal viruses to drop error rate • Approach is currently not optimal for short (< 3 kb) contigs • Plasmid sequences cannot be entirely distinguished from viruses: will be required the incorporation a dedicated tool for plasmid detection in VirSorter2 modular framework	https://github.com/jiarong/VirSorter2
VirFinder 2017 [44]	viral sequences prediction using machine learning based tool that uses k-mer frequencies **(ii)**	• k-mer frequency based, machine learning method - entirely avoids gene-based similarity searches; • have good performance, especially for shorter contigs (i.e., 1000 bp) • Independent of the input contig set composition: generates static prediction scores for each contig regardless of the other tested at the same time contigs; • Statistical framework: generates prediction scores, p values and q values showing the probability that sequence is a virus; this is useful for assessing and controlling the false positive rate • Eukaryotic DNA: not trained on eukaryotic DNA; may falsely identify eukaryotic sequences as phages • Additional filtering needed: users must filter out potential eukaryotic contamination before analysis • Limits of k-mer patterns: sensitive to the diversity of known viruses represented in the training sequence database; if viruses have some signal of k-mer patterns that are specific and not shared “universally” - the performance will be lower	https://github.com/jessieren/VirFinder
PPR-Meta 2019 [52]	3-class classifier for viral sequences prediction using novel neural network architecture – Bi-path Convolutional Neural Network (BiPathCNN) **(ii)**	• The first tool that can simultaneously identify phage and plasmid fragments • Innovation: the design of BiPathCNN, which Uses a more detailed method of characterizing DNA sequences - consider coding or non-coding region to improve the performance • Sequences length: BiPathCNN directly extracts sequence features from the raw data represented and may be less sensitive to sequence length • The recognition rate for prophages lower than that for phage contigs • Non-target organisms: trained only on prokaryote chromosomes, plasmids, and phages; other organisms not including in the training set - may affect the analysis results • Chimeric sequences: PPR-Meta cannot detailed judgments chimeras (e.g., prophages or chromosome chimeras)	https://github.com/zhenchengfang/PPR-Meta
Seeker 2020 [53]	viral sequences prediction using Long Short-Term Memory (LSTM) models **(ii)**	• Few parameters: uses relatively few parameters (152–212), reducing risk of memorization and overfitting • Short sequences: performs well on short sequences, important for metagenomic data • Low computational cost: requires minimal resources, runtime scales linearly with input length • Major advantages speed and maintains a high level of performance on viral sequences with little similarity to training data - and thus is well suited to discover new groups of phages. Classification errors: may misclassify some phage and bacterial genomes (does not perfectly distinguish phages from bacterial genomes) • Eukaryotic DNA: not trained on eukaryotic DNA; may falsely identify eukaryotic sequences as phages • Additional filtering needed: users must filter out potential eukaryotic contamination before analysis • Prophage detection: cannot reliably detect prophages in bacterial genomes - this feature is not supported	https://github.com/gussow/seeker
DeepVirFinder 2020 [45]	viral sequences prediction using convolutional neural networks (ConvNets) **(ii)**	• Prediction accuracy: extending the training data with additional viral sequences from environmental samples improves the prediction accuracy for under-represented viral groups • Prediction on short sequences: the tool is able to accurately classify even individual reads as viral - this can potentially simplify the complexity in assembly of viral genomes, improve assembly accuracy and reduce computing resources • Automatically feature engineering: unlike pre-defined feature-based machine learning models (k-mer frequencies logistic regression, gene-based random forest), DeepVirFinder automatically learned using a large number of training examples - this leads to higher accuracy due to the inclusion of hidden patterns • Eukaryotic DNA: not trained on eukaryotic DNA; may falsely identify eukaryotic sequences as phages • Additional filtering needed: users must filter out potential eukaryotic contamination before analysis	https://github.com/jessieren/DeepVirFinder
geNomad 2023 [55]	viruses and plasmids sequences prediction using a framework that combines information from gene content and a deep neural network **(i)+(ii)**	• High classification performance for viruses and plasmids (outperforms other tools in benchmarks), also provirus detection with high precision • Useful for metagenomic/metatranscriptomic assemblies • Integrated and Automated Workflow • The end-to-end command is user-friendly, as it runs the entire workflow • An active project: well-documented, and issues are addressed by the developers • High computational requirements, especially for memory (RAM) • Prediction on short sequences: perform poorly on contigs < 10 kb, due to their reliance on complete protein information • Conservativeness: tend to generate fewer false positives, but more true positives may be missed • Misclassification issues: tendency to incorrectly identify prokaryotic chromosomal sequences as plasmids	https://github.com/apcamargo/genomad

(i) reference-dependent approaches

(ii) reference-independent approaches

The resulting potential viral sequences can be evaluated using the CheckV tool (https://bitbucket.org/berkeleylab/checkv/src/master/), which is designed to assess the quality and completeness of viral genomes assembled from metagenomic data. Studies have shown that excluding contigs classified as ‘low-quality’ or ‘not-determined’ by CheckV assessment increases the consensus between independent phage prediction tools, highlighting a resulting set of high-confidence viral sequences. An additional advantage of the CheckV tool is that its database can be easily updated to include newly published viral genomes from the publicly available repositories [56, 57].

#### Prophages and the range of possible genome sizes for phages

Prophages are present in most bacterial taxa and play a crucial role in the evolution of prokaryotes [58–61]. They are often major sources of genetic diversity between closely related bacterial strains or pathovariants [62]. Prophages integration can significantly alter the phenotype of the bacterial host, facilitating the acquisition of new functions, including virulence, stress resistance, tolerance to phages and antimicrobials. Notably, many bacterial exotoxins involved in disease pathogenesis are encoded by prophages [63].

Prophages may contain so-called ‘accessory’ or ‘moron’ loci that are not required for the phage life cycle but influence host bacterial fitness, through a process known as lysogenic conversion. These loci are often highly expressed during lysogeny, while most phage-encoded genes are normally repressed at this stage. It has been hypothesized that accessory loci of prophages may possess unique transcriptional signatures distinct from lytic phage genes. However, identifying these genes solely on the basis of DNA sequence is very difficult [64, 65]. To clarify their functional role, approaches that include transcriptomic analysis are required.

In the context of studying the gut phageome, prophages represent a pivotal subject of analysis due to their predominance in the GV. However, annotating prophages in bacterial genomes and metagenomic data is associated with several methodological challenges. Over the past two decades, numerous bioinformatic tools have been developed to predict prophage regions in bacterial genomes [66]. Among these, VIBRANT [50], geNomad [55], DBSCAN-SWA [67], Phigaro [68], and CheckV [56] have demonstrated high prediction accuracy. Despite these advancements, many algorithms still face the difficulty of distinguishing between the bacterial chromosome and integrated prophage sequence, resulting in inaccurate predictions [10, 66]. Machine learning methods for prophage prediction are currently under active development, but their main limitation lies in the lack of manually annotated prophage regions in bacterial genomes. Such reference datasets are needed to test and train prophage region prediction algorithms and to improve the accuracy of prophage annotation. Several research groups are working toward the creation of such databases, which are expected to significantly improve the capabilities of modern prediction tools [66, 69–71].

Cryptic prophages – defined as prophages that cannot lyse their hosts and produce active phages – present an additional phage annotation challenge. These residual viral sequences can persist in the bacterial genome for many generations without functioning as true phages. This raises a classification dilemma: should these sequences be regarded as part of the bacterial genome, or as prophage because of their origin? Since temperate phages predominate in the GV, such classification difficulties pose a relevant problem [65, 72, 73]. It is also important to emphasize here that, contrary to the perception of them as a passive genetic cargo, cryptic prophages are capable of influencing the phenotype of the host bacterium. As a relatively permanent reservoir of genes, they provide the bacterium with numerous advantages for survival in competitive and unfavorable conditions. The influence of cryptic prophage genes on host cell physiology includes, for example, the acquisition of antibiotic resistance and tolerance, enhanced growth in rich and minimal media, as well as a strengthened host response to various environmental stresses, including oxidative and osmotic stress. Thus, these phage fossils significantly contribute to the metabolic potential of the host bacterium [74, 75].

**Fig. 1:**
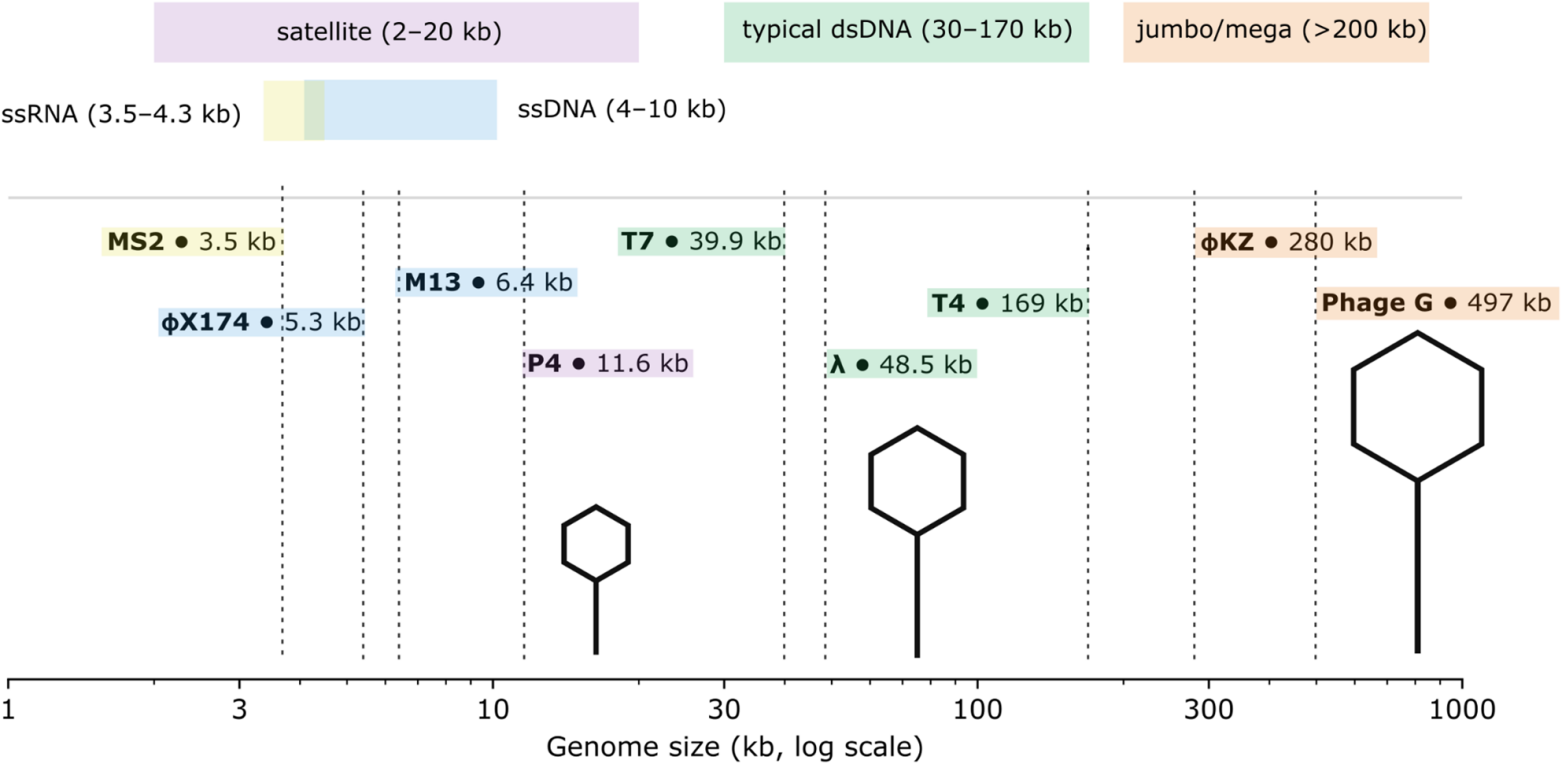
Comparative sizes of bacteriophage genomes. The following phages are shown in the figure: **MS2** – *Emesvirus zinderi* (GenBank: V00642); **phiX174** – *Escherichia* phage phiX174 (*Sinsheimervirus* phiX174, GenBank: J02482); **M13** – *Escherichia* phage M13 (*Inovirus* M13, GenBank: V00604); **P4** – *Enterobacteria* phage P4 (satellite phage P4, GenBank: NC_001609); **T7** – *Escherichia* phage T7 (*Teseptimavirus* T7, GenBank: V01146); **lambda** – *Escherichia* phage Lambda (*Lambdavirus lambda*, GenBank: J02459); **T4** – *Escherichia* phage T4 (Tequatrovirus T4, GenBank: AF158101.6); **phiKZ** – *Pseudomonas* phage phiKZ (*Phikzvirus* phiKZ, GenBank: AF399011); **phage G** – *Bacillus* phage G (*Donellivirus gee*, GenBank: JN638751)

When predicting viral sequences in metagenomic data, it is important to consider the range of possible phage genome sizes (Fig. 1). Metagenomic studies of various ecological niches have led to the discovery of the largest bacteriophages to date – the so-called megaphages – whose genomes can reach 500 kb or more [76–78]. The current record for the largest phage genome is a 735 kb megaphage from Lac Pavin, a freshwater meromictic crater lake in France (discovered through metagenomes sequenced from diverse ecosystems) [76]. Interestingly, some of megaphages use an alternative coding strategy. This poses a serious challenge for tools based on open reading frame homology searches with known viral genes, as recoded sequences are not recognized by standard annotation methods [79]. It should be noted here that recoding is also widespread among crAss-like phages and has been identified in a range of other phages [79–81]. On the other hand, emerging evidence suggests the existence of extremely small bacteriophages, with genome sizes well below the 10 kb threshold often used in metagenomic filtering [9, 10, 37, 56, 79]. Analysis of phage capsid structure by cryogenic electron microscopy (cryo-EM) revealed that icosahedral capsids can encapsulate DNA molecules of much smaller size. In addition, satellite phages – lacking the full set of genes necessary for independent replication and virion assembly – have been identified. These phages depend on co-infecting helper phages and may possess genomes as small as 5 kb (Fig. 1) [79].

Thus, the potential existence of both exceptionally large and unusually small phage genomes must be taken into account when analyzing metagenomic data. The use of methods focused solely on the traditional size of bacteriophages may result in the loss of a significant portion of viral diversity. This is especially important when developing new tools for identifying viral sequences, as genome size thresholds can significantly influence prediction outcomes.

#### Barriers to reliable phage genome assembly in metagenomics

At first glance, the obvious solution to the problem of identifying viral sequences in metagenomic data might seem to be assembling viral genomes directly from metagenomes, an approach known as viral metagenome-assembled genomes (vMAGs). This strategy relies on metagenomic binning, which clusters DNA sequences obtained from metagenomic sequencing into groups (bins) based on sequence similarity. In theory, such binning can enable the reconstruction of viral genomes. However, this approach faces a number of serious limitations that currently preclude its use as a reliable method.

First, viral sequences are often characterized by low and non-uniform coverage, which makes correctly assembling genomes difficult. Metagenomic binning tools are mainly focused on bacterial genomes, for which coverage is usually more stable and specific to different taxonomic groups. In contrast, for viruses, particularly temperate phages and rare virotypes – low coverage leads to fragmented assemblies and low binning accuracy [54, 65, 82].

Second, bacteriophage genomes exhibit a mosaic structure due to extensive horizontal gene transfer. Viral sequences, assembled in vMAGs, are often a concatenation of genome fragments from multiple unrelated phages, and represent chimeric assemblies. As a result, there are low-quality vMAGs that cannot be reliably classified [41].

Finally, genomes assembly from metagenomes produces many short contigs that are either discarded or incorrectly binned. This is particularly critical for viral genomes, because they often contain terminal repeat regions that are usually present in the assembly as short fragments. The loss of these regions can result in the omission of important portions of phage genomes [41].

Thus, at the current stage of development of computational methods and algorithms, viral binning tools cannot reliably reconstruct a complete, non-chimeric, and high-quality phage genome from metagenomic data.

#### Difficulties in taxonomic classification and host prediction

In addition to the techniques used for phage identification, the approaches and methods for their classification, including taxonomic systematization, remain a challenging task. The taxonomic classification of bacteriophages is more challenging than that of bacteria: bacteria possess universal marker genes (e.g., 16 S rRNA) which reflect their evolutionary relationships. By contrast, viruses lack any universally conserved genes on which a phylogeny could be constructed. Historically, phage classification was based on their morphology. The first taxonomic scheme was proposed by David Bradley in 1967 and focused on the best-studied phages of the time – tailed bacteriophages [83]. Later, in 1971, David Baltimore proposed classifying viruses based on the type of nucleic acid in virions, reflecting differences in viral replication strategies [84]. For a long time, the Baltimore scheme together with phage morphology, remained the basis for the bacteriophage classification.

With the advancement of molecular biology and sequencing technologies, it became evident that the genomic diversity of bacteriophages is much broader than previously recognized, particularly among tailed phages. It was discovered that there are significantly more uncultivated phages than cultured ones in natural ecosystems, leading to a significant expansion of bacteriophage taxonomy [85–88]. The resulting exponential growth in the number of new phage taxa has necessitated revising traditional classification systems.

In March 2022, the International Committee on Taxonomy of Viruses (ICTV, https://ictv.global/) approved a new classification system for tailed phages, abolishing the order *Caudovirales* and uniting all tailed phages into the class *Caudoviricetes*. New orders were introduced to replace the previous taxon and better reflect the origin and evolutionary relationships among of the various phage groups. Unfortunately, most of the viruses identified to date remain unclassified: among the ~ 55 500 records in the NCBI Virus portal (https://www.ncbi.nlm.nih.gov/labs/virus/vssi/#/, December 2025, host filter: Bacteria, taxid:2), ~ 25 000 (46%) do not have taxonomic annotation [88, 89].

Modern taxonomic classification of bacteriophages relies on a range of bioinformatic approaches, such as genome structure and organization analysis, proteomic analysis, clustering based on average nucleotide identity, and phylogenetic reconstruction. Accurate classification is complicated by the frequent genetic exchanges among phages, which can involve both individual genes and groups of genes. It is also important to consider, especially for phages with double-stranded DNA (dsDNA), extensive sharing of genes and/or gene cassettes – which require strict alignment criteria in their classification to avoid false-positive associations and artificial links between unrelated taxa. Another challenge of bioinformatic classification approaches is the rapid evolution of viral proteins.

With the development of metagenomics, it has begun to play an important role in the revelation of new phage taxa, allowing the identification of genomes of uncultured viruses. Parallel to this metagenomic studies have shown that a significant proportion of bacteriophages, particularly those infecting non-pathogenic bacteria, remain unclassified despite the existence of current classification systems.

In addition to the above, there are also the following limitations:

1) viral genome databases do not adequately represent the true diversity of viruses present in nature, because, for example there is a problem of overrepresentation of certain phage groups in reference databases. So, most studies of viromes show that they are dominated by tailed phages from the *Caudoviricetes* class, which is probably a consequence of the bias in databases towards the well-studied viruses. The problem of overrepresentation of the *Caudoviricetes* class in databases reduces the sensitivity of the tools to other groups of bacteriophages. Among the available tools, DeepVirFinder demonstrates the highest sensitivity in detecting a broad spectrum of phages, including understudied groups [3, 8, 45];

2) The viral taxonomy, as defined by the International Committee on Taxonomy of Viruses (ICTV), is constantly changing. Despite these challenges, recent years have seen the emergence of specialized tools for classifying viruses assembled from metagenomes, most of which rely on sequence similarity approaches, including BLAST-based or HMM-based searches [85]. These computational methods form the foundation for current efforts to systematically catalog viral diversity. A broader overview of classification approaches, algorithms, and software solutions is provided in two comprehensive reviews, discussing the state of the field and its development [85, 90].

Determining the phage host is one of the most challenging tasks in virology, especially for unculturable bacteriophages. Without reliable experimental methods to establish phage-host associations, researchers must rely on bioinformatic predictions. These predictions are usually based on molecular features indicating co-evolution and/or an arms race between phages and their hosts, identity with the genomes of reference viruses or hosts, as well as matches with CRISPR spacers encoded by the host and on sequence composition analysis [91].

Despite the development a number of tools to predict potential hosts of uncultured phages developed over the past decade (Table 2), the ability to associate newly identified phages with their hosts remains limited [92–101].

**Table 2:** An overview of phage host prediction tools

(bioRxiv)/ Year published	Tool/ reference	Host prediction approach	Features/notes
2016	**HostPhinder** [92]	predicts a phage’s host by k‑mers based genomic similarity to reference phages with known hosts	• virus-dependent (virus-virus similarity) • alignment-free • independent of gene prediction and host reference database • sequence composition-dependent tools similarity to at least one reference phage genome is required
2017	**WIsH** [93]	predicts a phage’s host by comparing k-mer composition between a query phage sequence and host genomes, via trains Markov models on each host and ranks hosts	• host-dependent (virus–host similarity) • alignment-free • independent of gene prediction and phage reference database sequence composition-dependent tools
(2019)/ 2022	**PHISDetector** [94]	predicts a phage’s host by integrative tool that detecting and integrating diverse in silico phage–host interaction signals (PHISs), and scoring their probability using machine learning models based on PHIS features	• virus–host alignment-free similarity (based on k-mer frequencies) • virus–host alignment-based similarity • virus–host CRISPR-based similarity • integrates additional features from putative prophage regions • integrates additional features from protein-protein interactions • use machine-learning approaches provides fancy visualizations for users
2020	**VirHostMatcher-Net** [95]	predicts a phage’s host by network-based, Markov random field framework, using integrate multiple alignment-free and alignment-based features	• virus-virus similarity • virus–host alignment-free similarity (based on k-mer frequencies) • virus–host alignment-based similarity • virus–host CRISPR-based similarity • use machine-learning approaches • improved accuracy and sensitivity by considering and integrating multiple signals integrative methods require a longer compute time since they need results from several individual prediction approaches
(2020)/ 2022	**vHULK** [96]	predicts a phage’s host by neural network-based tool based on protein-coding genes	• alignment-dependent • independ of host reference database • predicted protein sequences are affiliated to the pVOGs database • use two deep neural networks • high accuracy for phages related to known references required sharing at least one marker gene with a known phage reference
(2020)/ 2021	**RaFAH** [97]	predicts a phage’s host by machine-learning approach - Random Forest classifier based on similarity in protein content	• alignment-dependent/based • virus-dependent • based on protein content (using marker genes and HMM profiles) • for training database use host-dependent alignment-based methods • use machine-learning approaches only take mVCs as input
2021	**PredPHI** [98]	predicts a phage’s host by deep learning-based tool capable of predicting from sequence data	• alignment-free • use machine-learning approaches • convolutional neural network (CNN) • host proteomes dependent • informative features based on properties of protein sequences: - amino acid residue frequency - chemical composition molecular weight
2021	**Bacteriophage-Host-** **Prediction** [99]	predicts a phage’s host by machine-learning-based pipeline based on annotated receptor-binding protein (RBP) sequence data	• use machine-learning approaches • uses 218 features: - nucleotide/codon frequencies, codon usage bias and GC-content - relative abundance of amino acids - physicochemical properties of the sequences (protein length, molecular weight, isoelectric point, aromaticity, and others) - protein secondary structure (α-helix and β-sheet frequencies) - features describing protein sequences (composition, transition, and Z-scale) • model predicts bacterial hosts only for phages infecting *S. aureus*,* K. pneumoniae*,* A. baumannii*,* P. aeruginosa*,* E. coli*,* S. enterica* and *C. difficile*
2022	**CHERRY** [100]	predicts a phage’s host by formulates the host prediction problem as a link prediction in a multimodal graph; apply a graph convolutional encoder and use a two-layer neural network decoder to calculate the probability virus–host pair	• alignment-free • multimodal graph integrates different types of features (into the nodes and edges): - protein organization - CRISPR - sequence similarity - k-mer frequency (edges connect virus-host): - from labeled (training) and unlabeled (test) data
(2022)/ 2023	**iPHoP** [101]	predicts a phage’s host by integrate multiple approaches for host prediction and uses modular machine learning framework	• host-based and phage-based • integrative methods • run 6 host prediction approaches: - Blast - CRISPR - VirHostMatcher - WIsH - PHP - RaFAH • provides reliable host predictions at the genus rank because it relies on a suite of different tools, remains relatively slow compared to other tools

The advent of long-read metagenomic sequencing has provided the opportunities to overcome some of these limitations. By producing contiguous reads that can span entire prophages and their flanking host sequences, this technology can empirically link an integrated prophage with the genome of its host within complex communities like the gut microbiome, overcoming the challenges of fragmented short-read assemblies [102, 103].

Comparing spacers from bacterial CRISPR-Cas systems with phage genomes shows that most spacers have no matches, confirming the existence of a significant pool of ‘viral dark matter’. An additional problem is that the host ranges of phages have been studied extremely unevenly. Phages that infect well-studied pathogenic bacteria, such as *Escherichia coli* or *Klebsiella pneumoniae*, are much better described than phages infecting commensal or difficult to culture bacteria. This creates a substantial data bias, reducing the accuracy of predictions for rare or ecologically important microorganisms [104]. Therefore, the most effective strategy for phage-host prediction is integration of different computational approaches. For example, the iPHoP tool, which integrates host-based and phage-based approaches for reliable host taxonomy prediction, demonstrates good results on metagenomic data and is widely used [101]. Furthermore, developing large-scale datasets containing experimentally validated phage-host pairs also plays a crucial role in improving prediction accuracy [91]. As data accumulate and algorithms continue to improve, substantial progress in predicting phage-host relationships can be expected.

### Bacteriophages: promising therapeutic agents

**Fig. 2:**
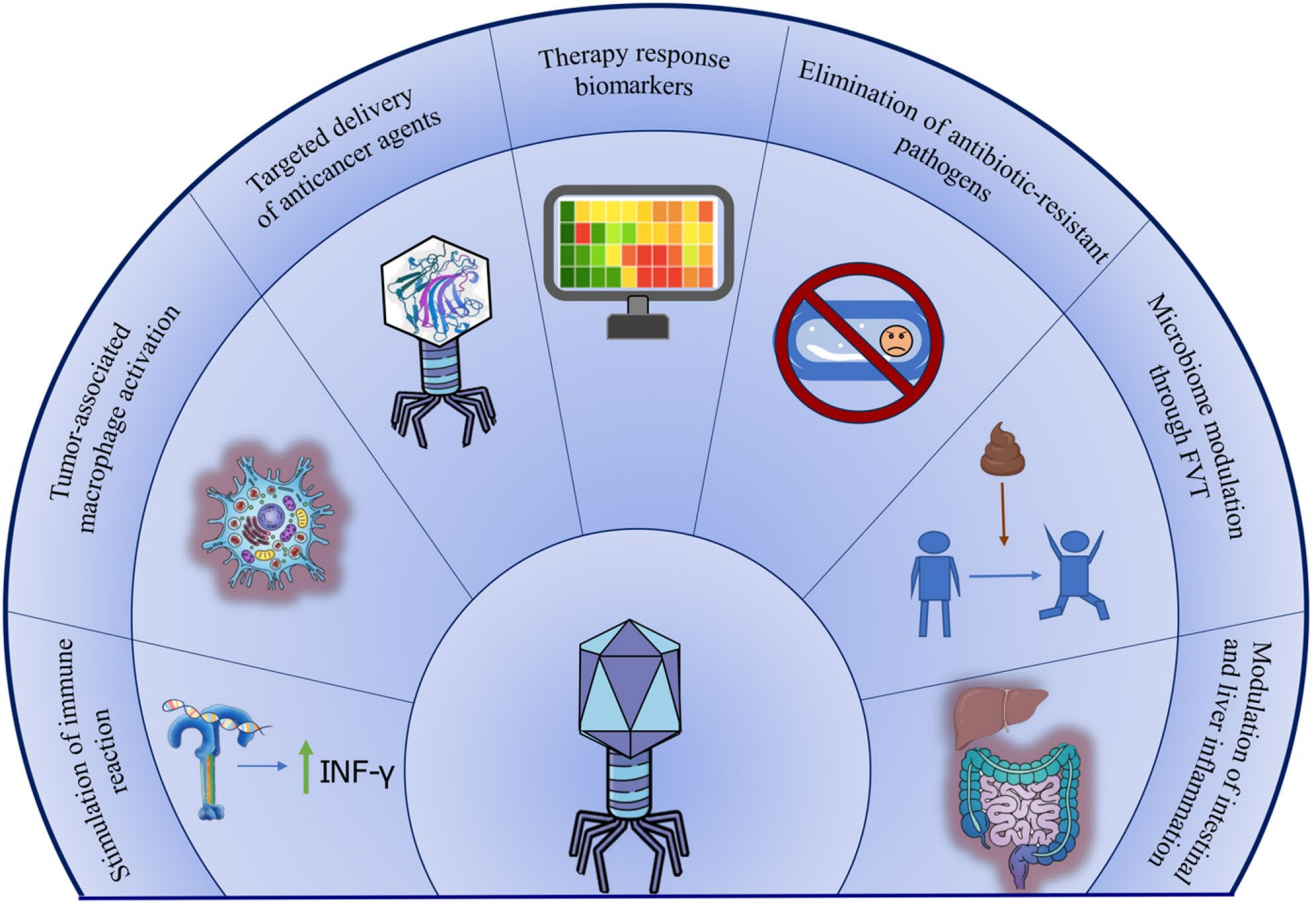
Therapeutic potential of bacteriophages

Bacteriophages are emerging as promising therapeutic tools with broad applications, extending beyond their classical use against antibiotic-resistant bacteria. Recent studies have highlighted their potential to modulate host immune responses, influence tumor progression, alleviate inflammatory and liver diseases, and serve as diagnostic and prognostic biomarkers in oncology (Fig. 2) [105, 106].

While phage therapy has traditionally targeted antibiotic-resistant pathogens, recent studies have begun exploring the potential of phages in oncology. Research into phage-based anticancer vaccines is underway, though none have yet received approval from the FDA or EMA [107]. Some studies suggest that phages can directly influence tumor cells. For example, phages T4 and HAP1 were shown to inhibit melanoma cell migration on fibronectin in vitro, indicating a potential role in limiting tumor dissemination [108]. Other studies have demonstrated that certain phages can activate tumor-associated macrophages, thereby promoting tumor cell destruction and stimulating innate immune responses [109]. Phages capable of modulating the immune reaction are also of growing interest. In germ-free mice, the administration of specific phages increased immune cell counts in the gut. For example, phages targeting *Lactobacillus*, *Escherichia*, and *Bacteroides* were shown to stimulate IFN-γ production via the TLR9 receptor, which detects viral DNA [21]. Moreover, engineered phages are being actively investigated for targeted delivery of anticancer agents and for immunostimulatory purposes. One study developed a modified T7 phage carrying a melanoma-targeting peptide and expressing the cytokine GM-CSF. Administration of this phage to mice resulted in 72% tumor growth suppression within 16 days and induced tumor infiltration by macrophages, dendritic cells, and CD8^+^ T-cells [110]. Another study engineered M13 phages expressing tumor-associated MAGE-A1 peptides, which elicited strong immune responses in mice, underscoring their potential in melanoma vaccine development [111]. Moreover, phage therapy has shown promise in treating hospital-acquired bacterial infections in cancer patients undergoing chemotherapy [112].

The development of inflammatory bowel diseases (IBD), including Crohn’s disease (CD) and ulcerative colitis (UC), is caused by the interaction of genetic, immunological and bacterial mechanisms. Numerous experimental models of enterocolitis support the immunological hypothesis of IBD pathogenesis, linking disease development to defects in both innate and adaptive immune responses. There is evidence of the influence of gut bacteriophages on the course of diseases associated with immune system disorders. Research indicates that patients with CD and UC exhibit reduced bacterial diversity in their GM, and the virome in these diseases differs from the norm and is characterized by a significant increase in the number of bacteriophages of the class *Caudoviricetes* (the original publication uses the now-obsolete order name *Caudovirales*) [113]. In addition, it is suggested that the virome plays an important role in the advancement of colorectal cancer (CRC). The increased alpha diversity of gut phages in CRC presumably leads to the depletion of the bacterial component of the GM due to an increase in the number of lytic infections, which in turn stimulates the development of CRC. An association with CRC has been shown for a number of viral families: in particular, enrichment of the *Myoviridae*,* Podoviridae*, and *Siphoviridae* families and depletion of *Herelleviridae* family (taxonomic names for virus families are given here according to the original cited works). The *Herelleviridae* family appears to promote the integrity of the gut barrier function, and its depletion in CRC potentially promotes tumor progression. Additionally, increased activity of fatty acid biosynthesis pathways has been identified in CRC patients, which has been previously shown to increase the risk of this disease [114, 115].

For liver disease, bacteriophages also have shown their therapeutic potential in pathologies, like alcoholic hepatitis. Transplanting bacteria from severe alcoholic hepatitis patients into humanized mice induced similar liver damage symptoms. It is known that alcoholic hepatitis has been linked to the presence of cytolysin-positive *Enterococcus faecalis* in the GM. Treatment with *E. faecalis* specific phages resulted in decreased levels of cytolysin in the liver, as well as decreased liver damage [116].

Another emerging therapeutic strategy is the fecal virome transplant (FVT), which involves the transfer of viral particles, including bacteriophages, and their associated metabolites from a healthy donor to a recipient. Unlike conventional fecal microbiota transplantation, this approach focuses specifically on the viral component of the GM. FVTs have already demonstrated clinical benefits in the treatment of recurrent *Clostridium difficile* infections in humans [117, 118], and preclinical studies suggest their potential to modulate obesity and metabolic alterations in mouse models [119]. Although still at an early stage of investigation, FVTs represent a promising avenue for harnessing the gut virome to restore microbial balance and influence host physiology. Other studies on FVT demonstrate its broad potential: in mice, FVT has been shown to modulate phenotypes (lean/obese) [120], improve metabolic status [119], and improve the proliferation of probiotic bacteria such as *Akkermansia muciniphila* [121]. Additionally, research in a yellow-feathered broilers using FVT has demonstrated that the virome and its metabolites, besides the microbiota, are also functional components contributing to the growth-promoting effect of fecal microbiota transplantation [122].

Importantly, an increasing number of publications suggest that intestinal bacteriophages may serve as biomarkers for various cancers, with potential diagnostic and prognostic applications. Predictive models based on gut bacteriophages have already demonstrated reproducibility and high predictive accuracy for colorectal cancer and colorectal adenoma [123, 124]. Furthermore, intestinal virus biomarkers have been shown to significantly distinguish patients with pancreatic cancer from healthy individuals, suggesting their potential utility in the early prognosis of the disease [125].

Together, these findings support the expanding therapeutic and diagnostic potential of gut phages, underscoring the need for further studies of the bacteriophage interaction mechanisms with both bacterial and host cells and its validation on large-scale clinical cohorts.

## Conclusion and perspectives

The human body is constantly exposed to large numbers of bacteriophages – an estimated 31 billion phage particles passing into the body daily [3, 18]. However, the impact of the gut phage community (gut phageome) on human health remains poorly understood, primarily due to challenges in virome isolating and sequencing, assembly, and interpretation.

Virome research, particularly in the context of the gut, is still in its early stages, with bioinformatic tools and standards actively evolving. Although efforts between 2016 and 2018 attempted to establish methodological guidelines on the most effective ways to analyze viruses in large metagenomic datasets – however, these remain insufficient [126–130]. Metagenomic studies of the GV typically provide qualitative rather than quantitative insights, identifying dominant phage groups, their possible expression strategies, and potential interactions with the host. It bears emphasis that the choice of bioinformatic tools and their parameters can significantly influence the results obtained [36]. To address this, the most promising seem to be either emerging hybrid or complex approaches, which combine complementary methods, like reference-dependent and independent tools, for example [79, 131]. As emphasized by Benler and Koonin in their article, that given the diversity of currently available tools, an integrated approach could make complete mapping of the global phageome a realistic task [79].

Beyond basic research, the therapeutic potential of bacteriophages is becoming increasingly evident. In addition to their established application against antibiotic-resistant bacteria, phages show promise in the treatment of inflammatory diseases, liver disorders and even in the treatment of cancer. Recent studies suggest that phages can affect not only bacterial communities but also directly interact with human immune cells, potentially influencing antitumor immunity. Nonetheless, many questions remain unresolved – including understanding the mechanisms of phage interaction with eukaryotic cells, the long-term effects of the GV on human health, and developing reliable predictive models to personalize phage therapy.

## Supplementary Information

Supplementary Material 1

## Data Availability

No datasets were generated or analysed during the current study.

## References

[1] Lozupone CA, Stombaugh JI, Gordon JI, Jansson JK, Knight R. Diversity, stability and resilience of the human gut microbiota. Nature. 2012;489(7415):220–30. 10.1038/nature11550. 22972295 10.1038/nature11550 PMC3577372

[2] Shkoporov AN, Clooney AG, Sutton TDS, Ryan FJ, Daly KM, Nolan JA, et al. The human gut virome is highly diverse, stable, and individual specific. Cell Host Microbe. 2019;26(4):527-541.e5. 10.1016/j.chom.2019.09.009. 31600503 10.1016/j.chom.2019.09.009

[3] Sutton TDS, Hill C. Gut bacteriophage: current understanding and challenges. Front Endocrinol (Lausanne). 2019;10:784. 10.3389/fendo.2019.00784. 31849833 10.3389/fendo.2019.00784 PMC6895007

[4] Krishnamurthy SR, Wang D. Origins and challenges of viral dark matter. Virus Res. 2017;239:136–42. 10.1016/j.virusres.2017.02.002. 28192164 10.1016/j.virusres.2017.02.002

[5] Roux S, Hallam SJ, Woyke T, Sullivan MB. Viral dark matter and virus-host interactions resolved from publicly available microbial genomes. Elife. 2015;4:e08490. 10.7554/eLife.08490. 26200428 10.7554/eLife.08490 PMC4533152

[6] Fitzgerald CB, Shkoporov AN, Upadrasta A, Khokhlova EV, Ross RP, Hill C. Probing the “Dark Matter” of the human gut phageome: culture assisted metagenomics enables rapid discovery and host-linking for novel bacteriophages. Front Cell Infect Microbiol. 2021;11:616918. 10.3389/fcimb.2021.616918. 33791236 10.3389/fcimb.2021.616918 PMC8005731

[7] Galperina A, Lugli GA, Milani C, De Vos WM, Ventura M, Salonen A, et al. The aggregated gut viral catalogue (AVrC): a unified resource for exploring the viral diversity of the human gut. PLoS Comput Biol. 2025;21(5):e1012268. 10.1371/journal.pcbi.1012268. 40315414 10.1371/journal.pcbi.1012268 PMC12068713

[8] Schackart KE, Graham JB, Ponsero AJ, Hurwitz BL. Evaluation of computational phage detection tools for metagenomic datasets. Front Microbiol. 2023;14:1078760. 10.3389/fmicb.2023.1078760. 36760501 10.3389/fmicb.2023.1078760 PMC9902911

[9] Camarillo-Guerrero LF, Almeida A, Rangel-Pineros G, Finn RD, Lawley TD. Massive expansion of human gut bacteriophage diversity. Cell. 2021;184(4):1098-1109.e9. 10.1016/j.cell.2021.01.029. 33606979 10.1016/j.cell.2021.01.029 PMC7895897

[10] Nayfach S, Páez-Espino D, Call L, Low SJ, Sberro H, Ivanova NN, et al. Metagenomic compendium of 189,680 DNA viruses from the human gut microbiome. Nat Microbiol. 2021;6(7):960–70. 10.1038/s41564-021-00928-6. 34168315 10.1038/s41564-021-00928-6 PMC8241571

[11] Mokili JL, Rohwer F, Dutilh BE. Metagenomics and future perspectives in virus discovery. Curr Opin Virol. 2012;2(1):63–77. 10.1016/j.coviro.2011.12.004. 22440968 10.1016/j.coviro.2011.12.004 PMC7102772

[12] Koonin EV, Dolja VV. A virocentric perspective on the evolution of life. Curr Opin Virol. 2013;3(5):546–57. 10.1016/j.coviro.2013.06.008. 23850169 10.1016/j.coviro.2013.06.008 PMC4326007

[13] Dutilh BE, Cassman N, McNair K, Sanchez SE, Silva GG, Boling L, et al. A highly abundant bacteriophage discovered in the unknown sequences of human faecal metagenomes. Nat Commun. 2014;5:4498. 10.1038/ncomms5498. 25058116 10.1038/ncomms5498 PMC4111155

[14] Aggarwala V, Liang G, Bushman FD. Viral communities of the human gut: metagenomic analysis of composition and dynamics. Mob DNA. 2017;8:12. 10.1186/s13100-017-0095-y. 29026445 10.1186/s13100-017-0095-y PMC5627405

[15] Chevallereau A, Pons BJ, van Houte S, Westra ER. Interactions between bacterial and phage communities in natural environments. Nat Rev Microbiol. 2022;20(1):49–62. 10.1038/s41579-021-00602-y. 34373631 10.1038/s41579-021-00602-y

[16] Broecker F, Moelling K. The roles of the virome in cancer. Microorganisms. 2021;9(12):2538. 10.3390/microorganisms9122538. 34946139 10.3390/microorganisms9122538 PMC8706120

[17] Shkoporov AN, Turkington CJ, Hill C. Mutualistic interplay between bacteriophages and bacteria in the human gut. Nat Rev Microbiol. 2022;20(12):737–49. 10.1038/s41579-022-00755-4. 35773472 10.1038/s41579-022-00755-4

[18] Nguyen S, Baker K, Padman BS, Patwa R, Dunstan RA, Weston TA, et al. Bacteriophage transcytosis provides a mechanism to cross epithelial cell layers. mBio. 2017;8(6):e01874-17. 10.1128/mBio.01874-17. 29162715 10.1128/mBio.01874-17 PMC5698557

[19] Carroll-Portillo A, Lin HC. Bacteriophage and the innate immune system: access and signaling. Microorganisms. 2019;7(12):625. 10.3390/microorganisms7120625. 31795262 10.3390/microorganisms7120625 PMC6956183

[20] Sinha A, Maurice CF. Bacteriophages: uncharacterized and dynamic regulators of the immune system. Mediators Inflamm. 2019;2019:3730519. 10.1155/2019/3730519. 31582898 10.1155/2019/3730519 PMC6754933

[21] Gogokhia L, Buhrke K, Bell R, Hoffman B, Brown DG, Hanke-Gogokhia C, et al. Expansion of bacteriophages is linked to aggravated intestinal inflammation and colitis. Cell Host Microbe. 2019;25(2):285-299.e8. 10.1016/j.chom.2019.01.008. 30763538 10.1016/j.chom.2019.01.008 PMC6885004

[22] Fujiki J, Schnabl B. Phage therapy: targeting intestinal bacterial microbiota for the treatment of liver diseases. JHEP Rep. 2023;5(12):100909. 10.1016/j.jhepr.2023.100909. 37965159 10.1016/j.jhepr.2023.100909 PMC10641246

[23] Fluckiger A, Daillère R, Sassi M, Sixt BS, Liu P, Loos F, et al. Cross-reactivity between tumor MHC class I-restricted antigens and an enterococcal bacteriophage. Science. 2020;369(6506):936–42. 10.1126/science.aax0701. 32820119 10.1126/science.aax0701

[24] Pham F, Moinard-Butot F, Coutzac C, Chaput N. Cancer and immunotherapy: a role for microbiota composition. Eur J Cancer. 2021;155:145–54. 10.1016/j.ejca.2021.06.051. 34375896 10.1016/j.ejca.2021.06.051

[25] Wu J, Chai T, Zhang H, Huang Y, Perry SW, Li Y, et al. Changes in gut viral and bacterial species correlate with altered 1,2-diacylglyceride levels and structure in the prefrontal cortex in a depression-like non-human primate model. Transl Psychiatry. 2022;12(1):74. 10.1038/s41398-022-01836-x. 35194021 10.1038/s41398-022-01836-x PMC8863841

[26] Mayneris-Perxachs J, Castells-Nobau A, Arnoriaga-Rodríguez M, Garre-Olmo J, Puig J, Ramos R, et al. Caudovirales bacteriophages are associated with improved executive function and memory in flies, mice, and humans. Cell Host Microbe. 2022;30(3):340-356.e8. 10.1016/j.chom.2022.01.013. 35176247 10.1016/j.chom.2022.01.013

[27] Liu C, Xing B, Li Z, Li J, Xiao M. A roadmap of isolating and investigating bacteriophage infecting human gut anaerobes. Essays Biochem. 2024;68(5):593–605. 10.1042/EBC20240116. 39611592 10.1042/EBC20240116 PMC11652169

[28] Shen J, Zhang J, Mo L, Li Y, Li Y, Li C, et al. Large-scale phage cultivation for commensal human gut bacteria. Cell Host Microbe. 2023;31(4):665-677.e7. 10.1016/j.chom.2023.03.013. 37054680 10.1016/j.chom.2023.03.013

[29] Zhao X, Sun C, Jin M, Chen J, Xing L, Yan J, et al. Enrichment culture but not metagenomic sequencing identified a highly prevalent phage infecting *Lactiplantibacillus plantarum* in human feces. Microbiol Spectr. 2023;11(3):e0434022. 10.1128/spectrum.04340-22. 36995238 10.1128/spectrum.04340-22 PMC10269749

[30] Guerin E, Shkoporov AN, Stockdale SR, Comas JC, Khokhlova EV, Clooney AG, et al. Isolation and characterisation of ΦcrAss002, a crAss-like phage from the human gut that infects Bacteroides xylanisolvens. Microbiome. 2021;9(1):89. 10.1186/s40168-021-01036-7. 33845877 10.1186/s40168-021-01036-7 PMC8042965

[31] Shkoporov AN, Khokhlova EV, Fitzgerald CB, Stockdale SR, Draper LA, Ross RP, et al. ΦCrAss001 represents the most abundant bacteriophage family in the human gut and infects Bacteroides intestinalis. Nat Commun. 2018;9(1):4781. 10.1038/s41467-018-07225-7. 30429469 10.1038/s41467-018-07225-7 PMC6235969

[32] Ogilvie LA, Jones BV. The human gut virome: a multifaceted majority. Front Microbiol. 2015;6:918. 10.3389/fmicb.2015.00918. 26441861 10.3389/fmicb.2015.00918 PMC4566309

[33] Hyman P. Phages for phage therapy: isolation, characterization, and host range breadth. Pharmaceuticals (Basel). 2019;12(1):35. 10.3390/ph12010035. 30862020 10.3390/ph12010035 PMC6469166

[34] Ackermann HW. Tailed bacteriophages: the order caudovirales. Adv Virus Res. 1998;51:135–201. 10.1016/s0065-3527(08)60785-x. 9891587 10.1016/S0065-3527(08)60785-X PMC7173057

[35] Reyes A, Haynes M, Hanson N, Angly FE, Heath AC, Rohwer F, Gordon JI. Viruses in the faecal microbiota of monozygotic twins and their mothers. Nature. 2010;466(7304):334–8. 10.1038/nature09199. 20631792 10.1038/nature09199 PMC2919852

[36] Ho SFS, Wheeler NE, Millard AD, van Schaik W. Gauge your phage: benchmarking of bacteriophage identification tools in metagenomic sequencing data. Microbiome. 2023;11(1):84. 10.1186/s40168-023-01533-x. 37085924 10.1186/s40168-023-01533-x PMC10120246

[37] Roux S, Emerson JB, Eloe-Fadrosh EA, Sullivan MB. Benchmarking viromics: an in silico evaluation of metagenome-enabled estimates of viral community composition and diversity. PeerJ. 2017;5:e3817. 10.7717/peerj.3817. 28948103 10.7717/peerj.3817 PMC5610896

[38] Gauthier CH, Hatfull GF. PhamClust: a phage genome clustering tool using proteomic equivalence. mSystems. 2023;8(5):e0044323. 10.1128/msystems.00443-23. 37791778 10.1128/msystems.00443-23 PMC10654103

[39] Hatfull GF, Hendrix RW. Bacteriophages and their genomes. Curr Opin Virol. 2011;1(4):298–303. 10.1016/j.coviro.2011.06.009. 22034588 10.1016/j.coviro.2011.06.009 PMC3199584

[40] Evseev P, Lukianova A, Sykilinda N, Gorshkova A, Bondar A, Shneider M, et al. *Pseudomonas* phage MD8: genetic mosaicism and challenges of taxonomic classification of lambdoid bacteriophages. Int J Mol Sci. 2021;22(19):10350. 10.3390/ijms221910350. 34638693 10.3390/ijms221910350 PMC8508860

[41] Mallawaarachchi V, Roach MJ, Decewicz P, Papudeshi B, Giles SK, Grigson SR, et al. Phables: from fragmented assemblies to high-quality bacteriophage genomes. Bioinformatics. 2023;39(10):btad586. 10.1093/bioinformatics/btad586. 37738590 10.1093/bioinformatics/btad586 PMC10563150

[42] Aytan-Aktug D, Grigorjev V, Szarvas J, Clausen PTLC, Munk P, Nguyen M, et al. SourceFinder: a Machine-Learning-Based tool for identification of Chromosomal, Plasmid, and bacteriophage sequences from assemblies. Microbiol Spectr. 2022;10(6):e0264122. 10.1128/spectrum.02641-22. 36377945 10.1128/spectrum.02641-22 PMC9769690

[43] Bai Z, Zhang YZ, Miyano S, Yamaguchi R, Fujimoto K, Uematsu S, et al. Identification of bacteriophage genome sequences with representation learning. Bioinformatics. 2022;38(18):4264–70. 10.1093/bioinformatics/btac509. 35920769 10.1093/bioinformatics/btac509 PMC9477532

[44] Ren J, Ahlgren NA, Lu YY, Fuhrman JA, Sun F. VirFinder: a novel k-mer based tool for identifying viral sequences from assembled metagenomic data. Microbiome. 2017;5(1):69. 10.1186/s40168-017-0283-5. 28683828 10.1186/s40168-017-0283-5 PMC5501583

[45] Ren J, Song K, Deng C, Ahlgren NA, Fuhrman JA, Li Y, et al. Identifying viruses from metagenomic data using deep learning. Quant Biol. 2020;8(1):64–77. 10.1007/s40484-019-0187-4. 34084563 10.1007/s40484-019-0187-4 PMC8172088

[46] Roux S, Enault F, Hurwitz BL, Sullivan MB. VirSorter: mining viral signal from microbial genomic data. PeerJ. 2015;3:e985. 10.7717/peerj.985. 26038737 10.7717/peerj.985 PMC4451026

[47] Jurtz VI, Villarroel J, Lund O, Voldby Larsen M, Nielsen M. MetaPhinder-Identifying bacteriophage sequences in metagenomic data sets. PLoS One. 2016;11(9):e0163111. 10.1371/journal.pone.0163111. 27684958 10.1371/journal.pone.0163111 PMC5042410

[48] Amgarten D, Braga LPP, da Silva AM, Setubal JC. MARVEL, a tool for prediction of bacteriophage sequences in metagenomic bins. Front Genet. 2018;9:304. 10.3389/fgene.2018.00304. 30131825 10.3389/fgene.2018.00304 PMC6090037

[49] Antipov D, Raiko M, Lapidus A, Pevzner PA. Metaviral spades: assembly of viruses from metagenomic data. Bioinformatics. 2020;36(14):4126–9. 10.1093/bioinformatics/btaa490. 32413137 10.1093/bioinformatics/btaa490

[50] Kieft K, Zhou Z, Anantharaman K. Vibrant: automated recovery, annotation and curation of microbial viruses, and evaluation of viral community function from genomic sequences. Microbiome. 2020;8(1):90. 10.1186/s40168-020-00867-0. 32522236 10.1186/s40168-020-00867-0 PMC7288430

[51] Guo J, Bolduc B, Zayed AA, Varsani A, Dominguez-Huerta G, Delmont TO, et al. Virsorter2: a multi-classifier, expert-guided approach to detect diverse DNA and RNA viruses. Microbiome. 2021;9(1):37. 10.1186/s40168-020-00990-y. 33522966 10.1186/s40168-020-00990-y PMC7852108

[52] Fang Z, Tan J, Wu S, Li M, Xu C, Xie Z, et al. Ppr-meta: a tool for identifying phages and plasmids from metagenomic fragments using deep learning. Gigascience. 2019;8(6):giz066. 10.1093/gigascience/giz066. 31220250 10.1093/gigascience/giz066 PMC6586199

[53] Auslander N, Gussow AB, Benler S, Wolf YI, Koonin EV. Seeker: alignment-free identification of bacteriophage genomes by deep learning. Nucleic Acids Res. 2020;48(21):e121. 10.1093/nar/gkaa856. 33045744 10.1093/nar/gkaa856 PMC7708075

[54] Shkoporov AN, Hill C. Bacteriophages of the human gut: the known unknown of the microbiome. Cell Host Microbe. 2019;25(2):195–209. 10.1016/j.chom.2019.01.017. 30763534 10.1016/j.chom.2019.01.017

[55] Camargo AP, Roux S, Schulz F, Babinski M, Xu Y, Hu B, et al. Identification of mobile genetic elements with genomad. Nat Biotechnol. 2024;42(8):1303–12. 10.1038/s41587-023-01953-y. 37735266 10.1038/s41587-023-01953-y PMC11324519

[56] Nayfach S, Camargo AP, Schulz F, Eloe-Fadrosh E, Roux S, Kyrpides NC. Checkv assesses the quality and completeness of metagenome-assembled viral genomes. Nat Biotechnol. 2021;39(5):578–85. 10.1038/s41587-020-00774-7. 33349699 10.1038/s41587-020-00774-7 PMC8116208

[57] McNair K, Bailey BA, Edwards RA. PHACTS, a computational approach to classifying the lifestyle of phages. Bioinformatics. 2012;28(5):614–8. 10.1093/bioinformatics/bts014. 22238260 10.1093/bioinformatics/bts014 PMC3289917

[58] Wendling CC. Prophage mediated control of higher order interactions-insights from multi-level approaches. Curr Opin Syst Biol. 2023;35:100469. 10.1016/j.coisb.2023.100469.

[59] Liao H, Liu C, Zhou S, Liu C, Eldridge DJ, Ai C, et al. Prophage-encoded antibiotic resistance genes are enriched in human-impacted environments. Nat Commun. 2024;15(1):8315. 10.1038/s41467-024-52450-y. 39333115 10.1038/s41467-024-52450-y PMC11437078

[60] Howard-Varona C, Hargreaves KR, Abedon ST, Sullivan MB. Lysogeny in nature: mechanisms, impact and ecology of temperate phages. ISME J. 2017;11(7):1511–20. 10.1038/ismej.2017.16. 28291233 10.1038/ismej.2017.16 PMC5520141

[61] López-Leal G, Camelo-Valera LC, Hurtado-Ramírez JM, Verleyen J, Castillo-Ramírez S, Reyes-Muñoz A. Mining of thousands of prokaryotic genomes reveals high abundance of prophages with a strictly narrow host range. mSystems. 2022;7(4):e0032622. 10.1128/msystems.00326-22. 35880895 10.1128/msystems.00326-22 PMC9426530

[62] Touchon M, Bernheim A, Rocha EP. Genetic and life-history traits associated with the distribution of prophages in bacteria. ISME J. 2016;10(11):2744–54. 10.1038/ismej.2016.47. 27015004 10.1038/ismej.2016.47 PMC5113838

[63] Casas V, Maloy S. Role of bacteriophage-encoded exotoxins in the evolution of bacterial pathogens. Future Microbiol. 2011;6(12):1461–73. 10.2217/fmb.11.124. 22122442 10.2217/fmb.11.124

[64] Owen SV, Canals R, Wenner N, Hammarlöf DL, Kröger C, Hinton JCD. A window into lysogeny: revealing temperate phage biology with transcriptomics. Microb Genom. 2020;6(2):e000330. 10.1099/mgen.0.000330. 32022660 10.1099/mgen.0.000330 PMC7067206

[65] Pargin E, Roach MJ, Skye A, Papudeshi B, Inglis LK, Mallawaarachchi V, et al. The human gut virome: composition, colonization, interactions, and impacts on human health. Front Microbiol. 2023;14:963173. 10.3389/fmicb.2023.963173. 37293229 10.3389/fmicb.2023.963173 PMC10244655

[66] Roach MJ, McNair K, Michalczyk M, Giles SK, Inglis LK, Pargin E, et al. Philympics 2021: prophage predictions perplex programs. F1000Res. 2022;10:758. 10.12688/f1000research.54449.2.

[67] Gan R, Zhou F, Si Y, Yang H, Chen C, Ren C, et al. DBSCAN-SWA: an integrated tool for rapid prophage detection and annotation. Front Genet. 2022;13:885048. 10.3389/fgene.2022.885048. 35518360 10.3389/fgene.2022.885048 PMC9061938

[68] Starikova EV, Tikhonova PO, Prianichnikov NA, Rands CM, Zdobnov EM, Ilina EN, Govorun VM. Phigaro: high-throughput prophage sequence annotation. Bioinformatics. 2020;36(12):3882–4. 10.1093/bioinformatics/btaa250. 32311023 10.1093/bioinformatics/btaa250

[69] Turner D, Adriaenssens EM, Tolstoy I, Kropinski AM. Phage annotation guide: guidelines for assembly and high-quality annotation. Phage (New Rochelle). 2021;2(4):170–82. 10.1089/phage.2021.0013. 35083439 10.1089/phage.2021.0013 PMC8785237

[70] Dahlman S, Avellaneda-Franco L, Rutten EL, Gulliver EL, Solari S, Chonwerawong M, et al. Isolation, engineering and ecology of temperate phages from the human gut. Nature. 2025;647(8090):698–705. 10.1038/s41586-025-09614-7. 41094135 10.1038/s41586-025-09614-7 PMC12629997

[71] Mavrich TN, Casey E, Oliveira J, Bottacini F, James K, Franz CMAP, et al. Characterization and induction of prophages in human gut-associated Bifidobacterium hosts. Sci Rep. 2018;8(1):12772. 10.1038/s41598-018-31181-3. 30143740 10.1038/s41598-018-31181-3 PMC6109161

[72] Sausset R, Petit MA, Gaboriau-Routhiau V, De Paepe M. New insights into intestinal phages. Mucosal Immunol. 2020;13(2):205–15. 10.1038/s41385-019-0250-5. 31907364 10.1038/s41385-019-0250-5 PMC7039812

[73] Hu J, Ye H, Wang S, Wang J, Han D. Prophage activation in the intestine: insights into functions and possible applications. Front Microbiol. 2021;12:785634. 10.3389/fmicb.2021.785634. 34966370 10.3389/fmicb.2021.785634 PMC8710666

[74] Wang X, Kim Y, Ma Q, Hong SH, Pokusaeva K, Sturino JM, et al. Cryptic prophages help bacteria cope with adverse environments. Nat Commun. 2010;1:147. 10.1038/ncomms1146. 21266997 10.1038/ncomms1146 PMC3105296

[75] Wang X, Wood TK. Cryptic prophages as targets for drug development. Drug Resist Updat. 2016;27:30–8. 10.1016/j.drup.2016.06.001. 27449596 10.1016/j.drup.2016.06.001

[76] Al-Shayeb B, Sachdeva R, Chen LX, Ward F, Munk P, Devoto A, et al. Clades of huge phages from across Earth’s ecosystems. Nature. 2020;578(7795):425–31. 10.1038/s41586-020-2007-4. 32051592 10.1038/s41586-020-2007-4 PMC7162821

[77] Michniewski S, Rihtman B, Cook R, Jones MA, Wilson WH, Scanlan DJ, et al. A new family of “megaphages” abundant in the marine environment. ISME Commun. 2021;1(1):58. 10.1038/s43705-021-00064-6. 37938293 10.1038/s43705-021-00064-6 PMC9723777

[78] Cook R, Crisci MA, Pye HV, Telatin A, Adriaenssens EM, Santini JM. Decoding huge phage diversity: a taxonomic classification of Lak megaphages. J Gen Virol. 2024;105(5):001997. 10.1099/jgv.0.001997. 38814706 10.1099/jgv.0.001997 PMC11165621

[79] Benler S, Koonin EV. Fishing for phages in metagenomes: what do we catch, what do we miss? Curr Opin Virol. 2021;49:142–50. 10.1016/j.coviro.2021.05.008. 34139668 10.1016/j.coviro.2021.05.008

[80] Yutin N, Benler S, Shmakov SA, Wolf YI, Tolstoy I, Rayko M, et al. Analysis of metagenome assembled viral genomes from the human gut reveals diverse putative CrAss-like phages with unique genomic features. Nat Commun. 2021;12:1044. 10.1038/s41467-021-21350-w. 33594055 10.1038/s41467-021-21350-w PMC7886860

[81] Ivanova NN, Schwientek P, Tripp HJ, Rinke C, Pati A, Huntemann M, et al. Stop codon reassignments in the wild. Science. 2014;344(6186):909–13. 10.1126/science.1250691. 24855270 10.1126/science.1250691

[82] Sutton TDS, Clooney AG, Ryan FJ, Ross RP, Hill C. Choice of assembly software has a critical impact on virome characterisation. Microbiome. 2019;7(1):12. 10.1186/s40168-019-0626-5. 30691529 10.1186/s40168-019-0626-5 PMC6350398

[83] Bradley DE. Ultrastructure of bacteriophage and bacteriocins. Bacteriol Rev. 1967;31(4):230–314. 10.1128/br.31.4.230-314.1967. 4865539 10.1128/br.31.4.230-314.1967 PMC408286

[84] Baltimore D. Viral genetic systems. Trans N Y Acad Sci. 1971;33(3):327–32. 10.1111/j.2164-0947.1971.tb02600.x. 4327120 10.1111/j.2164-0947.1971.tb02600.x

[85] Andrade-Martínez JS, Camelo Valera LC, Chica Cárdenas LA, Forero-Junco L, López-Leal G, Moreno-Gallego JL, et al. Computational tools for the analysis of uncultivated phage genomes. Microbiol Mol Biol Rev. 2022;86(2):e0000421. 10.1128/mmbr.00004-21. 35311574 10.1128/mmbr.00004-21 PMC9199400

[86] Li X, Cheng R, Zhang C, Shao Z. Genomic diversity of phages infecting the globally widespread genus *Sulfurimonas*. Commun Biol. 2024;7(1):1428. 10.1038/s42003-024-07079-4. 39488617 10.1038/s42003-024-07079-4 PMC11531552

[87] Camargo AP, Nayfach S, Chen IA, Palaniappan K, Ratner A, Chu K, et al. IMG/VR v4: an expanded database of uncultivated virus genomes within a framework of extensive functional, taxonomic, and ecological metadata. Nucleic Acids Res. 2023;51(D1):D733–43. 10.1093/nar/gkac1037. 36399502 10.1093/nar/gkac1037 PMC9825611

[88] Roux S, Mutalik VK. Tapping the treasure trove of atypical phages. Curr Opin Microbiol. 2024;82:102555. 10.1016/j.mib.2024.102555. 39388759 10.1016/j.mib.2024.102555

[89] Turner D, Kropinski AM, Adriaenssens EM. A roadmap for genome-based phage taxonomy. Viruses. 2021;13(3):506. 10.3390/v13030506. 33803862 10.3390/v13030506 PMC8003253

[90] Zhu Y, Shang J, Peng C, Sun Y. Phage family classification under Caudoviricetes: a review of current tools using the latest ICTV classification framework. Front Microbiol. 2022;13:1032186. 10.3389/fmicb.2022.1032186. 36590402 10.3389/fmicb.2022.1032186 PMC9800612

[91] Coclet C, Roux S. Global overview and major challenges of host prediction methods for uncultivated phages. Curr Opin Virol. 2021;49:117–26. 10.1016/j.coviro.2021.05.003. 34126465 10.1016/j.coviro.2021.05.003

[92] Villarroel J, Kleinheinz KA, Jurtz VI, Zschach H, Lund O, Nielsen M, et al. HostPhinder: a phage host prediction tool. Viruses. 2016;8(5):116. 10.3390/v8050116. 27153081 10.3390/v8050116 PMC4885074

[93] Galiez C, Siebert M, Enault F, Vincent J, Söding J. WIsH: who is the host? Predicting prokaryotic hosts from metagenomic phage contigs. Bioinformatics. 2017;33(19):3113–4. 10.1093/bioinformatics/btx383. 28957499 10.1093/bioinformatics/btx383 PMC5870724

[94] Zhou F, Gan R, Zhang F, Ren C, Yu L, Si Y, et al. PHISDetector: a tool to detect diverse in silico phage-host interaction signals for virome studies. Genomics Proteomics Bioinf. 2022;20(3):508–23. 10.1016/j.gpb.2022.02.003. 10.1016/j.gpb.2022.02.003 PMC9801046 35272051

[95] Wang W, Ren J, Tang K, Dart E, Ignacio-Espinoza JC, Fuhrman JA, et al. A network-based integrated framework for predicting virus-prokaryote interactions. NAR Genomics Bioinform. 2020;2(2):lqaa044. 10.1093/nargab/lqaa044. 10.1093/nargab/lqaa044 PMC7324143 32626849

[96] Amgarten D, Iha BKV, Piroupo CM, da Silva AM, Setubal JC. vHULK, a new tool for bacteriophage host prediction based on annotated genomic features and neural networks. PHAGE. 2022;3(4):204–12. 10.1089/phage.2021.0016. 36793881 10.1089/phage.2021.0016 PMC9917316

[97] Coutinho FH, Zaragoza-Solas A, López-Pérez M, Barylski J, Zielezinski A, Dutilh BE, et al. RaFAH: host prediction for viruses of bacteria and archaea based on protein content. Patterns. 2021;2(7):100274. 10.1016/j.patter.2021.100274. 34286299 10.1016/j.patter.2021.100274 PMC8276007

[98] Li M, Wang Y, Li F, Zhao Y, Liu M, Zhang S, et al. A deep learning-based method for identification of bacteriophage-host interaction. IEEE ACM Trans Comput Biol Bioinform. 2021;18(5):1801–10. 10.1109/TCBB.2020.3017386. 32813660 10.1109/TCBB.2020.3017386 PMC8703204

[99] Boeckaerts D, Stock M, Criel B, Gerstmans H, De Baets B, Briers Y. Predicting bacteriophage hosts based on sequences of annotated receptor-binding proteins. Sci Rep. 2021;11(1):1467. 10.1038/s41598-021-81063-4. 33446856 10.1038/s41598-021-81063-4 PMC7809048

[100] Shang J, Sun Y. CHERRY: a computational method for accurate prediction of virus-pRokarYotic interactions using a graph encoder-decoder model. Brief Bioinform. 2022;23(5):bbac182. 10.1093/bib/bbac182. 35595715 10.1093/bib/bbac182 PMC9487644

[101] Roux S, Camargo AP, Coutinho FH, Dabdoub SM, Dutilh BE, Nayfach S, et al. iPHoP: an integrated machine learning framework to maximize host prediction for metagenome-derived viruses of archaea and bacteria. PLoS Biol. 2023;21(4):e3002083. 10.1371/journal.pbio.3002083. 37083735 10.1371/journal.pbio.3002083 PMC10155999

[102] Wirbel J, Hickey AS, Chang D, Enright NJ, Dvorak M, Chanin RB, et al. Long-read metagenomics reveals phage dynamics in the human gut Microbiome. Nature. 2025. 10.1038/s41586-025-09786-2. 41299176 10.1038/s41586-025-09786-2 PMC12823448

[103] Lai S, Wang H, Bork P, Chen WH, Zhao XM. Long-read sequencing reveals extensive gut phageome structural variations driven by genetic exchange with bacterial hosts. Sci Adv. 2024;10(33):eadn3316. 10.1126/sciadv.adn3316. 39141729 10.1126/sciadv.adn3316 PMC11323893

[104] Glickman C, Hendrix J, Strong M. Simulation study and comparative evaluation of viral contiguous sequence identification tools. BMC Bioinformatics. 2021;22(1):329. 10.1186/s12859-021-04242-0. 34130621 10.1186/s12859-021-04242-0 PMC8207588

[105] Kabwe M, Dashper S, Tucci J. The microbiome in pancreatic cancer-implications for diagnosis and precision bacteriophage therapy for this low survival disease. Front Cell Infect Microbiol. 2022;12:871293. 10.3389/fcimb.2022.871293. 35663462 10.3389/fcimb.2022.871293 PMC9160434

[106] Shen S, Huo D, Ma C, Jiang S, Zhang J. Expanding the colorectal cancer biomarkers based on the human gut phageome. Microbiol Spectr. 2021;9(3):e0009021. 10.1128/Spectrum.00090-21. 34935421 10.1128/Spectrum.00090-21 PMC8693921

[107] Roehnisch T, Then C, Nagel W, Blumenthal C, Braciak T, Donzeau M, et al. Phage idiotype vaccination: first phase I/II clinical trial in patients with multiple myeloma. J Transl Med. 2014;12:119. 10.1186/1479-5876-12-119. 24885819 10.1186/1479-5876-12-119 PMC4113220

[108] Dabrowska K, Skaradziński G, Jończyk P, Kurzepa A, Wietrzyk J, Owczarek B, et al. The effect of bacteriophages T4 and HAP1 on in vitro melanoma migration. BMC Microbiol. 2009;9:13. 10.1186/1471-2180-9-13. 19154575 10.1186/1471-2180-9-13 PMC2639589

[109] Eriksson F, Tsagozis P, Lundberg K, Parsa R, Mangsbo SM, Persson MA, et al. Tumor-specific bacteriophages induce tumor destruction through activation of tumor-associated macrophages. J Immunol. 2009;182(5):3105–11. 10.4049/jimmunol.0800224. 19234207 10.4049/jimmunol.0800224

[110] Hwang YJ, Myung H. Engineered bacteriophage T7 as a potent anticancer agent in vivo. Front Microbiol. 2020;11:491001. 10.3389/fmicb.2020.491001. 33072000 10.3389/fmicb.2020.491001 PMC7541933

[111] Brišar N, Šuster K, Brezar SK, Vidmar R, Fonović M, Cör A. An engineered M13 filamentous nanoparticle as an antigen carrier for a malignant melanoma immunotherapeutic strategy. Viruses. 2024;16(2):232. 10.3390/v16020232. 38400008 10.3390/v16020232 PMC10893169

[112] Szczaurska-Nowak K, Dabrowska K, Celka M, Kurzepa A, Nevozhay D, Wietrzyk J, et al. Antitumor effect of combined treatment of mice with cytostatic agents and bacteriophage T4. Anticancer Res. 2009;29(6):2361–70. 19528503

[113] Norman JM, Handley SA, Baldridge MT, Droit L, Liu CY, Keller BC, et al. Disease-specific alterations in the enteric virome in inflammatory bowel disease. Cell. 2015;160(3):447–60. 10.1016/j.cell.2015.01.002. 25619688 10.1016/j.cell.2015.01.002 PMC4312520

[114] Zuo W, Michail S, Sun F. Metagenomic analyses of multiple gut datasets revealed the association of phage signatures in colorectal cancer. Front Cell Infect Microbiol. 2022;12:918010. 10.3389/fcimb.2022.918010. 35782128 10.3389/fcimb.2022.918010 PMC9240273

[115] Hannigan GD, Duhaime MB, Ruffin MT 4th, Koumpouras CC, Schloss PD. Diagnostic potential and interactive dynamics of the colorectal cancer virome. mBio. 2018;9(6):e02248-18. 10.1128/mBio.02248-18. 30459201 10.1128/mBio.02248-18 PMC6247079

[116] Duan Y, Llorente C, Lang S, Brandl K, Chu H, Jiang L, et al. Bacteriophage targeting of gut bacterium attenuates alcoholic liver disease. Nature. 2019;575(7783):505–11. 10.1038/s41586-019-1742-x. 31723265 10.1038/s41586-019-1742-x PMC6872939

[117] Ott SJ, Waetzig GH, Rehman A, Moltzau-Anderson J, Bharti R, Grasis JA, et al. Efficacy of sterile fecal filtrate transfer for treating patients with Clostridium difficile infection. Gastroenterology. 2017;152(4):799-811.e7. 10.1053/j.gastro.2016.11.010. 27866880 10.1053/j.gastro.2016.11.010

[118] Yu Y, Wang W, Zhang F. The next generation fecal microbiota transplantation: to transplant bacteria or virome. Adv Sci. 2023;10(35):e2301097. 10.1002/advs.202301097. 10.1002/advs.202301097 PMC10724401 37914662

[119] Mao X, Larsen SB, Zachariassen LSF, Brunse A, Adamberg S, Mejia JLC, et al. Transfer of modified gut viromes improves symptoms associated with metabolic syndrome in obese male mice. Nat Commun. 2024;15(1):4704. 10.1038/s41467-024-49152-w. 38830845 10.1038/s41467-024-49152-w PMC11148109

[120] Borin JM, Liu R, Wang Y, Wu TC, Chopyk J, Huang L, et al. Fecal virome transplantation is sufficient to alter fecal microbiota and drive lean and obese body phenotypes in mice. Gut Microbes. 2023;15(1):2236750. 10.1080/19490976.2023.2236750. 37475473 10.1080/19490976.2023.2236750 PMC10364654

[121] Rasmussen TS, Mentzel CMJ, Danielsen MR, Jakobsen RR, Zachariassen LSF, Castro Mejia JL, et al. Fecal virome transfer improves proliferation of commensal gut *Akkermansia muciniphila* and unexpectedly enhances the fertility rate in laboratory mice. Gut Microbes. 2023;15(1):2208504. 10.1080/19490976.2023.2208504. 37150906 10.1080/19490976.2023.2208504 PMC10167882

[122] Chen S, Liu T, Chen J, Shen H, Wang J. Fecal virome transplantation confirms non-bacterial components (virome and metabolites) participate in fecal microbiota transplantation-mediated growth performance enhancement and intestinal development in broilers with spatial heterogeneity. Microorganisms. 2025;13(8):1795. 10.3390/microorganisms13081795. 40871301 10.3390/microorganisms13081795 PMC12388568

[123] Tamayo-Trujillo R, Guevara-Ramírez P, Cadena-Ullauri S, Paz-Cruz E, Ruiz-Pozo VA, Zambrano AK. Human virome: implications in cancer. Heliyon. 2023;9(3):e14086. 10.1016/j.heliyon.2023.e14086. 36873548 10.1016/j.heliyon.2023.e14086 PMC9957661

[124] Chen F, Li S, Guo R, Song F, Zhang Y, Wang X, et al. Meta-analysis of fecal viromes demonstrates high diagnostic potential of the gut viral signatures for colorectal cancer and adenoma risk assessment. J Adv Res. 2023;49:103–14. 10.1016/j.jare.2022.09.012. 36198381 10.1016/j.jare.2022.09.012 PMC10334131

[125] Zhang P, Shi H, Guo R, Li L, Guo X, Yang H, et al. Metagenomic analysis reveals altered gut virome and diagnostic potential in pancreatic cancer. J Med Virol. 2024;96(7):e29809. 10.1002/jmv.29809. 39016466 10.1002/jmv.29809

[126] Pratama AA, Bolduc B, Zayed AA, Zhong ZP, Guo J, Vik DR, et al. Expanding standards in viromics: in silico evaluation of dsDNA viral genome identification, classification, and auxiliary metabolic gene curation. PeerJ. 2021;9:e11447. 10.7717/peerj.11447. 34178438 10.7717/peerj.11447 PMC8210812

[127] Paez-Espino D, Eloe-Fadrosh EA, Pavlopoulos GA, Thomas AD, Huntemann M, Mikhailova N, et al. Uncovering Earth’s virome. Nature. 2016;536(7617):425–30. 10.1038/nature19094. 27533034 10.1038/nature19094

[128] Paez-Espino D, Pavlopoulos GA, Ivanova NN, Kyrpides NC. Nontargeted virus sequence discovery pipeline and virus clustering for metagenomic data. Nat Protoc. 2017;12(8):1673–82. 10.1038/nprot.2017.063. 28749930 10.1038/nprot.2017.063

[129] Dutilh BE, Reyes A, Hall RJ, Whiteson KL. Editorial: virus discovery by metagenomics: the (im)possibilities. Front Microbiol. 2017;8:1710. 10.3389/fmicb.2017.01710. 28943867 10.3389/fmicb.2017.01710 PMC5596103

[130] Emerson JB, Roux S, Brum JR, Bolduc B, Woodcroft BJ, Jang HB, et al. Host-linked soil viral ecology along a permafrost thaw gradient. Nat Microbiol. 2018;3(8):870–80. 10.1038/s41564-018-0190-y. 30013236 10.1038/s41564-018-0190-y PMC6786970

[131] Marquet M, Hölzer M, Pletz MW, Viehweger A, Makarewicz O, Ehricht R, et al. What the phage: a scalable workflow for the identification and analysis of phage sequences. Gigascience. 2022;11:giac110. 10.1093/gigascience/giac110. 36399058 10.1093/gigascience/giac110 PMC9673492

