# Correction: Bacteriophages in gut metagenomes: from analysis to application

**DOI:** 10.1186/s12985-026-03170-w

**Published:** 2026-04-28

**Authors:** Natalia Zakharevich, Aleksandra Strokach, Egor Shitikov, Ksenia Klimina

**Affiliations:** 1 Department of Biomedicine and Genomics, Lopukhin Federal Research and Clinical Center of Physical-Chemical Medicine of Federal Medical Biological Agency, Moscow, Russia; 2 Department of Immunology, Faculty of Biology, Lomonosov Moscow State University, Moscow, Russia

**Correction to: Virology Journal (2026) 23:40**

10.1186/s12985-026-03069-6

In the original version of this article [1], the given and family names of authors Zakharevich Natalia, Strokach Aleksandra, Shitikov Egor and Klimina Ksenia were incorrectly structured. The name was displayed correctly in all versions at the time of publication. The citation of this article has been corrected to Zakharevich, N., Strokach, A., Shitikov, E. and Klimina, K.

The original article has been corrected.
