# Supplementary material for: Bacteriophages in gut metagenomes: from analysis to application
Source: Virol J. 2026 Jan 22;23:40. doi: 10.1186/s12985-026-03069-6 (PMC12911206; doi:10.1186/s12985-026-03069-6)
Supplement: Supplementary file 1 — Supplementary Material 1 [file 12985_2026_3069_MOESM1_ESM.docx]

SUPPLEMENTARY TABLE

**Supplementary Table 1.** Description and characteristics of viral/bacteriophage databases

| **Database** | **Description** | **Content and Notes** | **link** |
| --- | --- | --- | --- |
| *General virus/phage genome portal* | | | |
| **NCBI Virus** | portal for viral sequence data from RefSeq, GenBank and other NCBI repositories | RefSeq/Genbank/EMBL/DDBJ (these databanks exchange its data on a daily basis) contains around 7169 phage sequences; nucleotide/protein sequences (including complete genomes), genomic and functional annotations, virus taxonomy, host distribution and other metadata; Blast available | <https://www.ncbi.nlm.nih.gov/labs/virus/>  <https://www.ddbj.nig.ac.jp/index-e.html>  <https://www.ebi.ac.uk/> |
| *Phage-focused genome databases* | | | |
| **PhagesDB** [1] | interactive database of phages that infect bacterial hosts within the phylum *Actinobacteria* | - contains around 3754 phage sequences - actinobacteriophage sequences with phenotype annotated, hosts, Phams, metadata and others - data curated - NCBI/GenBank cross-links - Blast available | https://phagesdb.org/ |
| **PhageScope** [2] | online phage database with comprehensive annotations | - curated PhageScope database includes around 874000 phage sequences - stores phage sequences from multiple public repositories and published datasets - provides completeness assessment, taxonomic annotation, structural annotation, functional annotation, host range and lifestyle information - provides genome comparison, automatic analyses and visualizations for curated phages | https://phagescope.deepomics.org/ |
| **PhageDive** [3] | comprehensive database for prokaryotic viruses containing data dispersed across multiple sources, like scientific publications, specialized databases or internal files of culture collections | - to date PhageDive contains around 1200 phages from three public collections (DSMZ, Félix d'Hérelle Reference Centre for Bacterial Viruses and NCTC) - provides search function from the sections like taxonomy or morphology, experimental data (host range, genomic data, etc.) and available metadata (e.g. geographical origin, isolation source) - PhageDive is interoperable with other resources – NCBI, the Viral Host Range database (VHRdb) of Institute Pasteur, BacDive and MediaDive databases of DSMZ - an important feature is the link between experimental data, the culture collection number and the repository of the corresponding physical bioresource | https://phagedive.dsmz.de/ |
| *Large integrated viral catalogs* | | | |
| **IMG/VR** [4] | provides access to the collection of viral sequences obtained from (meta)genomes, along with functional annotation and rich metadata | - contains around 177361 phage sequences - nucleotide/protein sequences, functional annotation, virus taxonomy, host prediction and other metadata - a web interface enables users to efficiently search viruses based on genome features and/or sequence similarity - viral sequences are complemented with genome quality estimation using CheckV | <https://img.jgi.doe.gov/>cgi-bin/vr/main.cgi <https://genome.jgi.doe.gov/portal/>IMG_VR/IMG_VR.home.html |
| **MGV: Metagenomic Gut Virus catalog** [5] | metagenomic gut virus catalogue from publicly available human stool metagenomes | - comprises 189680 viral draft genomes from 11810 bulk metagenomes from human stool samples derived from 61 previously published studies - genomes estimate to be >50% complete and representing 54118 candidate viral species | https://portal.nersc.gov/MGV https://github.com/snayfach/MGV |
| **GPD: Gut Phage Database** [6] | gut phage database - high-quality, large-scale catalog of phage genomes | - database contains 142809 non-redundant gut phage genomes from 28060 metagenomes and 2898 reference genomes of cultured gut bacteria - host assignment, epidemiology analysis, analysis of the global distribution of phages - curated database - includes over 40000 high-quality genomes with a median size of 47.68 kb - clade *Gubaphage*, is one of a highly prevalent clade in the human gut, was discovered and described, the *Gubaphage* clade was found in 5 continents | https://www.sanger.ac.uk/data/gut-phage-database/ https://github.com/cai91/GPD |
| **GVD: Gut Virome Database** [7] | human gut virome database | - contains 33242 unique viral populations (approximately species-level taxa) from 2697 viral particle or microbial metagenomes from 1986 individuals representing 16 countries (32 studies) - taxonomy, host prediction - limitations: geographic and ethnic representation across the dataset is not very broad, not all available human gut and other human-associated bulk metagenomic datasets were analysed, given the current, the extent of RNA viruses in the human gut is likely underestimated | <https://bitbucket.org/MAVERICLab/gvd/src/master/>  https://datacommons.cyverse.org/browse/iplant/home/shared/iVirus/Gregory_and_Zablocki_GVD_Jul2020 |
| *Protein families/HMMs for phage annotation* | | | |
| **PHROG** [8] | prokaryotic virus remote homologous groups database | - contains 38880 PHROGs (protein orthologous groups) containing 868340 proteins from complete genomes of viruses infecting bacteria or archaea, in addition to 12498 curated prophages derived from cultivated microbial isolates - standardized annotation was attributed to each PHROG - website provides access to: prokaryotic virus genomes and see its taxonomy, list of proteins, genomic map, etc…   all PHROGs and see its annotation, list of proteins, multiple alignment, etc…   - sequences, multiple alignments, HMM profiles and annotations are downloadable as zipped archives on this website - users can compare their protein datasets to the PHROG profiles | https://phrogs.lmge.uca.fr/ |
| **VOGDB** [9] | database of virus orthologous groups | - VOGDB release 231 (2025-08-02), 692869 viral proteins were clustered and produced 48445 VOGs, 39624 VFAMs and 33073 VFOLDs (number of genomes 15019) - VOGDB is a multi-layer database:   *first layer* is based on pair-wise sequence similarities,  *second layer* is based on the sequence profile alignments,  *third layer* uses predicted protein structures to find the most remote similarity   - uses all virus genomes from RefSeq and partially reannotates them; VOGDB is updated with every RefSeq release - limitations:   VOGDB is based on the annotations provided by the underlying RefSeq database (VOGDB addresses the problem of inconsistent and outdated annotation by filtering and partial reannotation in order to ensure a higher quality of final clusters) | https://vogdb.org/ |
| *Virus–host linkage/taxonomy resources/viral classification* | | | |
| **Virus-Host DB** [10] | comprehensive and manually curated database of taxonomic links between viruses and their cellular hosts | - database covers viruses with complete genomes stored in   1) NCBI/RefSeq and 2) GenBank whose accession numbers are listed in EBI Genomes or INPHARED   - the host information is collected from RefSeq, GenBank (in free text format), UniProt, ViralZone, and manually curated with additional information obtained by literature surveys | https://www.genome.jp/virushostdb/ |
| **ICTV Taxonomy** | taxonomy browser | - official taxonomic resources - taxonomy browser provides access to the current virus taxonomy – page updates whenever a new taxonomy release has been approved by the ICTV - *release v2*, August 22, 2025:   7 realms, 11 kingdoms, 22 phyla, 49 classes, 93 orders, 368 families, 3768 genera, 16213 species | https://ictv.global/taxonomy |
| **ViPTree**  [11] | web server provided through GenomeNet to generate viral proteomic trees for classification of viruses based on genome-wide similarities | - ViPTree server generates a “proteomic tree” of viral genome sequences based on genome-wide sequence similarities computed by tBLASTx - the proteomic tree approach is effective to investigate genomes of both newly sequenced viruses and identified in metagenomes - ViPTree offers an alignment visualization useful for comparative genomics of viruses | https://github.com/yosuken/ViPTreeGen?ysclid=mf2muw83d7667503255  https://www.genome.jp/viptree |

**References:**

1. Russell DA, Hatfull GF. PhagesDB: the actinobacteriophage database. Bioinformatics. 2017;33(5):784-786. doi: 10.1093/bioinformatics/btw711

2. Wang RH, Yang S, Liu Z, Zhang Y, Wang X, Xu Z, et al. PhageScope: a well-annotated bacteriophage database with automatic analyses and visualizations. Nucleic Acids Res. 2024;52(D1):D756-D761. doi: 10.1093/nar/gkad97

3. Rolland C, Wittmann J, Reimer LC, Sardà Carbasse J, Schober I, Dudek CA, et al. PhageDive: the comprehensive strain database of prokaryotic viral diversity. Nucleic Acids Res. 2025;53(D1):D819-D825. doi: 10.1093/nar/gkae878

4. Camargo AP, Nayfach S, Chen IA, Palaniappan K, Ratner A, Chu K, et al. IMG/VR v4: an expanded database of uncultivated virus genomes within a framework of extensive functional, taxonomic, and ecological metadata. Nucleic Acids Res. 2023;51(D1):D733-D743. doi: 10.1093/nar/gkac1037

5. Nayfach S, Páez-Espino D, Call L, Low SJ, Sberro H, Ivanova NN, et al. Metagenomic compendium of 189,680 DNA viruses from the human gut microbiome. Nat Microbiol. 2021;6(7):960-970. doi: 10.1038/s41564-021-00928-6

6. Camarillo-Guerrero LF, Almeida A, Rangel-Pineros G, Finn RD, Lawley TD. Massive expansion of human gut bacteriophage diversity. Cell. 2021;184(4):1098-1109.e9. doi: 10.1016/j.cell.2021.01.029

7. Gregory AC, Zablocki O, Zayed AA, Howell A, Bolduc B, Sullivan MB. The Gut Virome Database Reveals Age-Dependent Patterns of Virome Diversity in the Human Gut. Cell Host Microbe. 2020;28(5):724-740.e8. doi: 10.1016/j.chom.2020.08.003

8. Terzian P, Olo Ndela E, Galiez C, Lossouarn J, Pérez Bucio RE, Mom R, Toussaint A, Petit MA, et al. PHROG: families of prokaryotic virus proteins clustered using remote homology. NAR Genom Bioinform. 2021;3(3):lqab067. doi: 10.1093/nargab/lqab067

9. Trgovec-Greif L, Hellinger HJ, Mainguy J, Pfundner A, Frishman D, Kiening M, et al. VOGDB-Database of Virus Orthologous Groups. Viruses. 2024;16(8):1191. doi: 10.3390/v16081191

10. Mihara T, Nishimura Y, Shimizu Y, Nishiyama H, Yoshikawa G, Uehara H, et al. Linking Virus Genomes with Host Taxonomy. Viruses. 2016;8(3):66. doi: 10.3390/v8030066

11. Nishimura Y, Yoshida T, Kuronishi M, Uehara H, Ogata H, Goto S. ViPTree: the viral proteomic tree server. Bioinformatics. 2017;33(15):2379-2380. doi: 10.1093/bioinformatics/btx15
